# Next-generation sequencing identifies HOXA6 as a novel oncogenic gene in low grade glioma

**DOI:** 10.18632/aging.203977

**Published:** 2022-03-29

**Authors:** Jiang Xiulin, Chunyan Wang, Jishu Guo, Chenyang Wang, Chenglong Pan, Zhi Nie

**Affiliations:** 1 Department of Pathology, First Affiliated Hospital of Kunming Medical University, Kunming 650032, Yunnan, China; 2 Department of Neurology, First Affiliated Hospital of Kunming Medical University, Kunming 650032, Yunnan, China; 3 Yunnan Province Clinical Research Center for Neurological Diseases, Kunming 650032, Yunnan, China; 4 Key Laboratory of Animal Models and Human Disease Mechanisms of Chinese Academy of Sciences, Kunming Institute of Zoology, Kunming 650223, Yunnan, China; 5 Institute for Ecological Research and Pollution Control of Plateau Lakes, School of Ecology and Environmental Science, Yunnan University, Kunming 650500, Yunnan, China

**Keywords:** next-generation sequencing, low grade glioma, HOXA family, prognostic biomarker, cell migration

## Abstract

Background: Low grade glioma is one of the most common lethal cancers in the human nervous system. Emerging evidence has demonstrated that homeobox A cluster (HOXA) gene family plays a critical role in the transcriptional regulation as well as cancer initiation and progression. However, the expression, biological functions and upstream regulatory mechanism of 11 HOXAs in low grade glioma are not yet clear.

Methods: In this study, we utilized various public databases and bioinformatics analyzed, including TCGA, CGGA, Rembrandt, HPA, LinkedOmics, cBioPortal, TISDIB, single-sample GSEA (ssGSEA), TIMER, LnCeVar, LASSO regression, Cox regression, Kaplan-Meier plot, and receiver operating, characteristic (ROC) analyses, GDSC and CTRP databases to analyzed the mRNA and protein expression profiles, gene mutation, clinical features, diagnosis, prognosis, signaling pathway, TMB, immune subtype, immune cell infiltration, immune modulator, ceRNA network and drug sensitivity of 11 HOXAs. Growth curve and transwell assays were utilized to study the biological characteristics of HOXA6 in LGG progression.

Results: In the present study, we found that 11 HOXAs (HOXA1, HOXA2, HOXA3, HOXA4, HOXA5, HOXA6, HOXA7, HOXA9, HOXA10, HOXA11 and HOXA3) were consistently up-regulated in LGG tissues and GBM tissues. Up-regulated of the HOXAs expression were significantly correlated with higher tumor stage, IDH mutation status, 1p/19q co-deletion, histological type and primary therapy outcome. Survival analyses showed that higher expression of HOXA1, HOXA2, HOXA3, HOXA4, HOXA5, HOXA7, HOXA9, HOXA10, HOXA11 and HOXA13 were correlated with shorter overall survival (OS), disease-specific survival (DSS) and progression-free survival (PFS) in LGG patients. Univariate and multivariate analyses revealed that HOXA1, HOXA6 expression and tumor grade, age, primary therapy outcome and age were independent factors affecting the prognosis of LGG patients. ROC curve analysis of HOXAs showed that HOXAs had a high accuracy (AUC > 0.80) in predicting LGG. Furthermore, gene functional enrichment analysis indicated that HOXAs mainly involved in the inflammatory response and immune regulation signaling pathway. CNV and DNA methylation significantly affect the expression of HOXAs. Finally, we uncover that HOXAs expression are highly correlated with immune cells infiltrate, immune modulator and drug sensitivity. We also uncover that the HOXAs related ceRNA network in LGG. More importantly, we found that HOXA6 was highly expressed in LGG cells lines and significantly affected their proliferation and migration abilities.

Conclusions: In conclusion, our data demonstrated that HOXA was correlated with progression and immune infiltration, and could serve as a prognostic biomarker for LGG.

## INTRODUCTION

Low grade glioma (LGG) is a relatively common tumor in the central nervous system that mainly includes World Health Organization (WHO) grade 2 and 3 gliomas [1]. Emerging evidence has demonstrated that the molecular characteristics of gliomas including mutated isocitrate dehydrogenase 1 and 2 genes (IDH1/2) and co-deletion of 1p/19q [2]. With various advances in earlier diagnosis and newer therapies have increased overall survival, disparities in access to and outcomes of care for low grade glioma persist. Recently, molecular biomarkers have been indicated that to be helpful in the diagnosis and prognosis of various cancers. Therefore, uncovering the molecular mechanisms underlying initiation and progression of LGG and identifying highly reliable biomarkers is crucial to improve the diagnosis and treatment of LGG patients.

As a member of homeobox genes, the HOXA cluster genes family including the HOXA1 to HOXA7, HOXA9, HOXA10, HOXA11 and HOXA13 [3], The protein encoded by HOXA cluster genes family were contain the DNA-binding homeobox motif. Accumulating evidence demonstrated that HOXA genes family plays a central role in transcription regulation and cancer initiation and progression. For example, it has been showed that HOXA1 was highly expressed in breast cancer and its high expression was correlated with poor prognosis and tumor progression of BRCA [4]. Zheng et al. found that HOXA5 by inhibits the Wnt/β-catenin pathway and repress the proliferation and neoplasia of cervical cancer cells [5]. Furthermore, HOXA7 was increased in HCC. Depletion of HOXA7 by inhibits Snail expression and inhibits HCC metastatic [6]. Boman et al. found that elevate the expression of HOXA4 and HOXA9 promotes the self-renewal of colon cancer stem cell [7]. However, the expression profiles, genetic alterations, clinicopathological parameters, diagnosis values, prognostic values and immune functions of 11 HOXAs in low grade glioma remains to be further elucidated.

In this study, we conducted a comprehensive bioinformatics analysis of the expression profiles, genetic alterations, clinicopathological parameters, diagnosis values, prognostic values and immune functions of HOXA family in LGG. Furthermore, we perform CNV, DNA methylation, KEGG analysis for the HOXA family. Finally, we examined the correlation between HOXAs expression and immune cells infiltration, immune modulator and drug sensitivity. We also perform the *in vitro* assay to explore the function of HOXAs in LGG progression.

## MATERIALS AND METHODS

### Analysis the expression, clinical information and prognosis of HOXAs

We utilized TCGA database (https://www.cancer.gov/) and CGGA (http://www.cgga.org.cn/) [8–10] to examine the expression, clinical information and prognosis of HOXAs in LGG. HPA (https://www.proteinatlas.org/) database utilized to examine the protein of HOXAs in LGG [11].

### Function analysis for HOXAs in LGG

In the present research, we utilized the LinkedOmics database (http://www.linkedomics.org/login.php) obtained the co-expression genes of HOXAs in LGG. We were using clusterProfiler package to examine the function of HOXAS in LGG [12, 13]. GeneMANIA database (http://genemania.org/) used to constructed gene-gene interaction network of HOXAs [14].

### Cox regression analysis and Kaplan-Meier survival analysis

We utilized cox regression analysis to examine the correlation between HOXAs expression and overall survival and disease-specific survival of patients using the TCGA databases. The Kaplan-Meier method was used to assess the difference between high and low risk groups based on the best separation of HOXAs expression, employing R packages of survminer and survival.

### Immune infiltration analysis

TIMER (https://cistrome.shinyapps.io/timer/) [15], an interactive web portal, could perform comprehensive analysis on the infiltration levels of different immune cells. In this study, we were using the TIMER database to explore the correlation between HOXAs and diverse immune cell infiltration in LGG. We also were using GSVA R package to quantify the LGG immune infiltration of 24 tumor-infiltrating immune cells in tumor samples via ssGSEA [16]. The TISIDB (http://cis.hku.hk/TISIDB/) database utilized analysis the expression of HOXAs in different immune subtype of LGG [17].

### Analysis the correlation between the HOXAs expression and drug sensitivity

The Genomics of Drug Sensitivity in Cancer (GDSC) database (http://www.cancerRxgene.org) is the largest public resource for information on drug sensitivity in cancer cells and molecular markers of drug response. Data are freely available without restriction. GDSC currently contains drug sensitivity data for almost 75 000 experiments, describing response to 138 anticancer drugs across almost 700 cancer cell lines. In this study, we utilized the Genomics of Drug Sensitivity in Cancer (GDSC) (http://www.cancerRxgene.org) and the Cancer Therapeutics Response Portal (http://www.broadinstitute.org/ctrp) databases to analysis the correlation between HOXAs expression and drug sensitivity [18, 19]. Spearman’s rank correlation was employed to measure the correlation between HOXAs expression and diverse drug sensitivity.

### Gene mutation analysis

The cBioPortal database (http://www.cbioportal.org/) and GSCA (http://bioinfo.life.hust.edu.cn/web/GSCALite/) used to analysis the gene mutation, DNA methylation of HOXAs in LGG [20, 21].

### LncRNA/miRNA/mRNA network construction

To explore the potential upstream regulatory of HOXAs in LGG, we used the LnCeVar database (http://www.bio-bigdata.net/LnCeVar/index.jsp)constructed a competing endogenous RNA (ceRNA) network [22].

### Cell culture conditions

GBM cells lines (including A172, SF295, T98G and U251 cells) were purchased from cell bank of Kunming Institute of Zoology, and cultured in DMEM medium (Corning) supplemented with 10% fetal bovine serum (FBS) and 1% penicillin/streptomycin at 37° C in atmosphere containing 95% air and 5% CO2.

### Quantitative real-time PCR

The qRT-PCR assay was performed as documented [23]. The primer sequences are list follows: HOXA6-F: TCCCGGACAAGACGTACAC, HOXA6-R: CGCCACTGAGGTCCTTATCA. B-actin-F: CTTCGCGGGCGACGAT, β-actin-R: CCATAGGAATCCTTCTGACC. The expression quantification was obtained with the 2^−ΔΔCt^ method.

### Cell proliferation assay

Cell proliferation was performed as previously documented [24]. Briefly, for cell proliferation assay, indicated cells were plated into 12-well plates at a density of 1.5×10^4^, the cell numbers were subsequently counted each day using an automatic cell analyzer Countstar (Shanghai Ruiyu Biotech Co., China, IC1000). (ns, p ≥ 0.05; *, p < 0.05; **, p < 0.01; ***, p < 0.001).

### Cell migration assay

Cell migration assays was performed as previously documented [24]. Briefly, to produce a wound, the monolayer cells in 6-well plate were scraped in a straight line with pipette tips. Plate was then washed with PBS to remove detached cells. Photographs of the scratch were taken at indicated time points using Nikon inverted microscope (Ti-S).

### Statistical analysis

Correlation analysis was performed using Pearson correlation test. Kaplan-Meier survival curves were plotted to exhibit the overall survival for LGG patients. Univariate and multivariate Cox regression analyses were used to examine the independent prognostic significance of each variable enrolled in this finding. The significance of the data between two experimental groups was determined by Student’s t-test and multiple group comparisons were analyzed by one-way ANOVA. P < 0.05 (*), P < 0.01 (**) and P < 0.001 (***), were considered significant.

## RESULTS

### HOXAs were highly expressed in LGG

**Figure 1:**
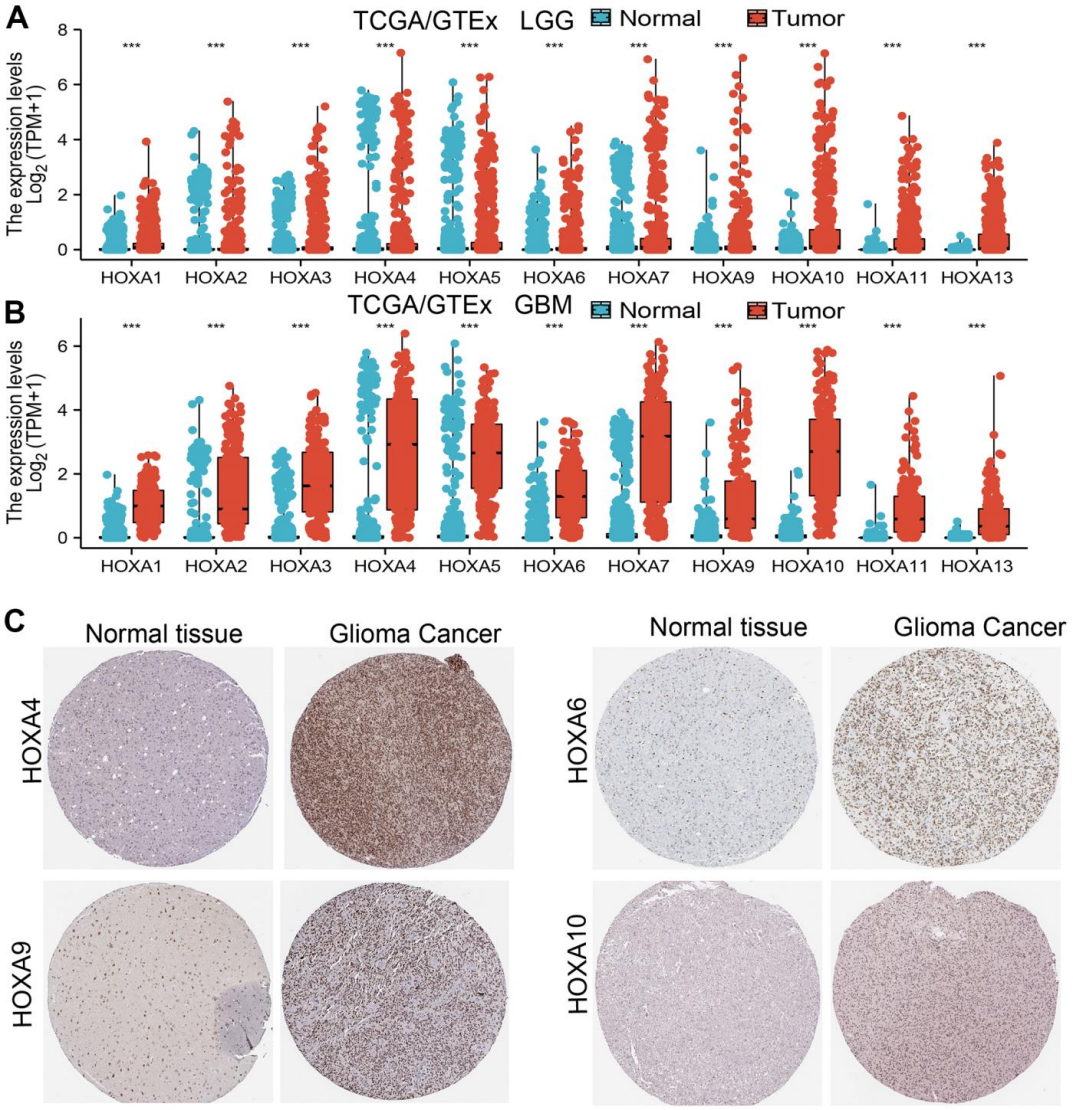
**HOXAs is highly expressed in LGG.** (**A**, **B**) The expression of HOXAs in LGG and GBM examine by TCGA/GTEx databases. (**C**) The expression of HOXAs in LGG tissue examine by HPA database.

We used TCGA and GTEx databases explored the expression of 11 HOXAs in GBM and LGG, the results showed that 11 HOXAs were significantly higher in lower-grade glioma (LGG) and glioblastoma multiforme (GBM) than those in normal samples (Figure 1A, 1B). Furthermore, we uncover that the protein of HOXA4, HOXA6, HOXA9 and HOXA10 were highly expressed in LGG tissue compared with control group based on the Human Protein Atlas datasets (Figure 1C). Collectively, these results suggested that HOXAs were overexpressed in LGG tissues.

### Correlation between HOXAs expression level and clinicopathological characteristics of patients with LGG

**Figure 2:**
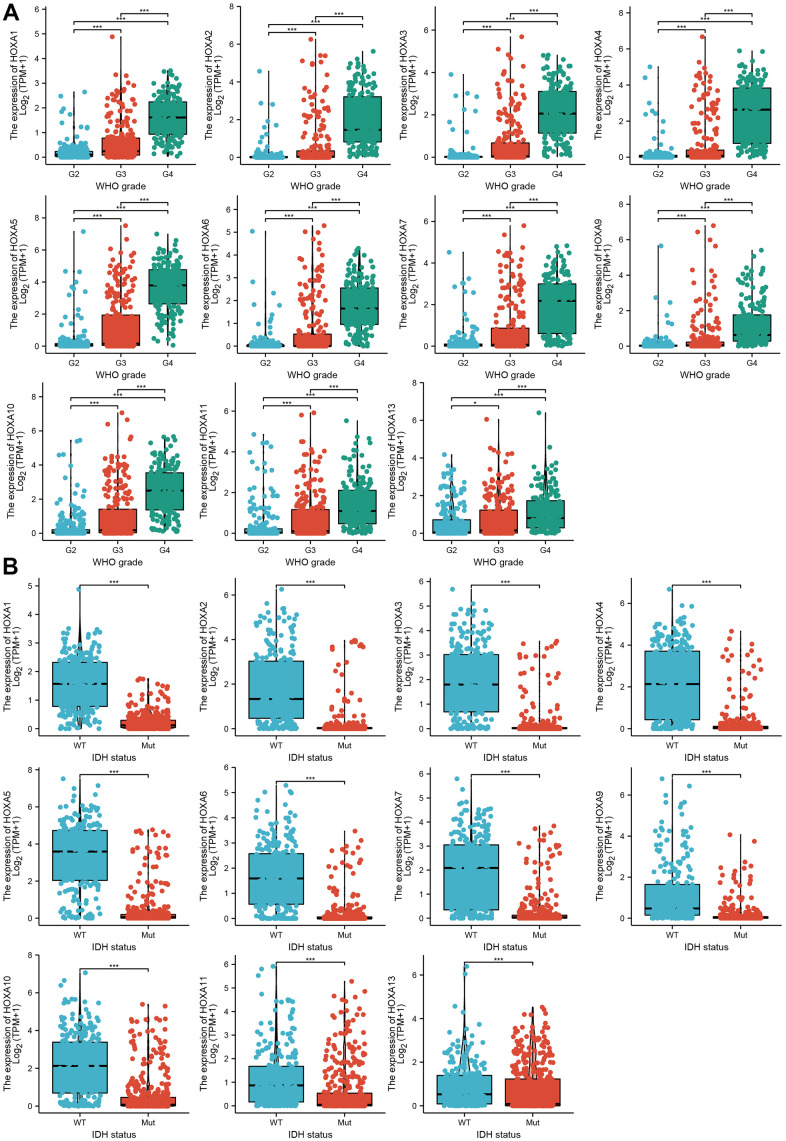
**The correlation between HOXAs expression and clinical information in LGG.** (**A**, **B**) The correlation between HOXAs expression and clinical features, including the higher tumor grades and IDH mutation status in glioma based on TCGA-LGG. *p < 0.05, **p < 0.01, ***p < 0.001.

**Figure 3:**
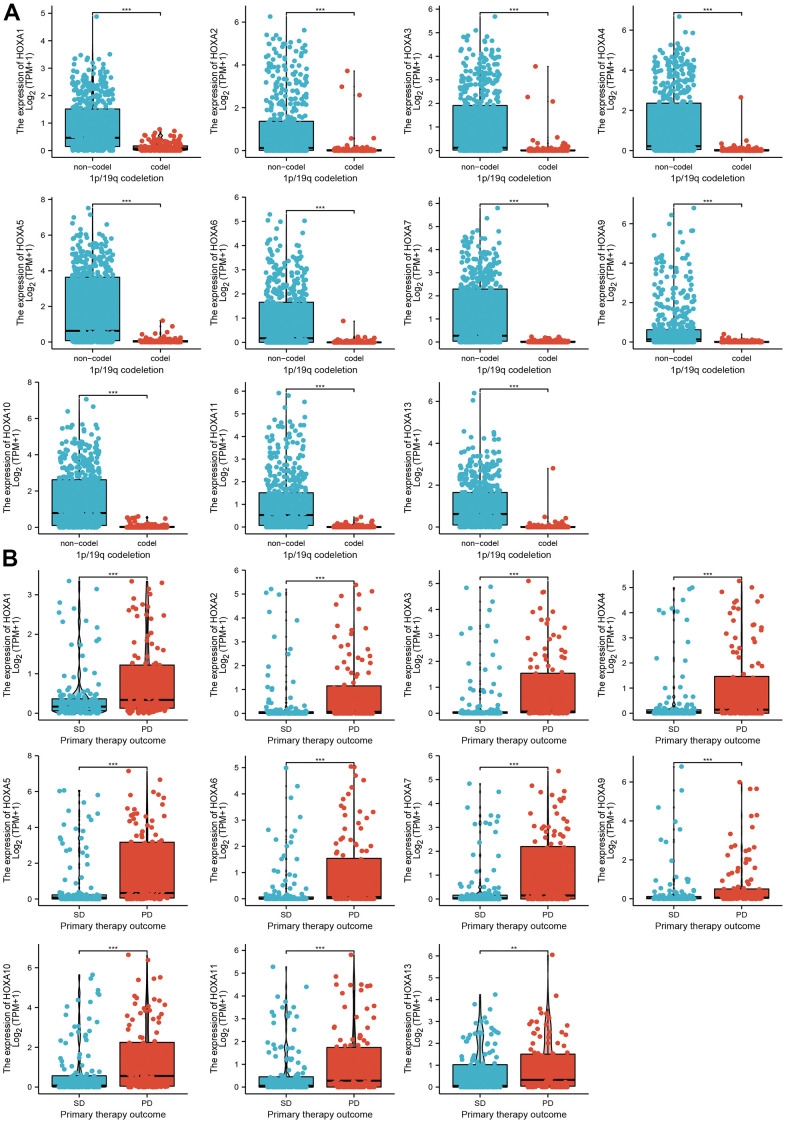
**The correlation between HOXAs expression and clinical information in LGG.** (**A**, **B**) The correlation between HOXAs expression and clinical features, including the 1p/19q codeletion and primary therapy outcome in glioma based on TCGA-LGG. *p < 0.05, **p < 0.01, ***p < 0.001.

**Figure 4:**
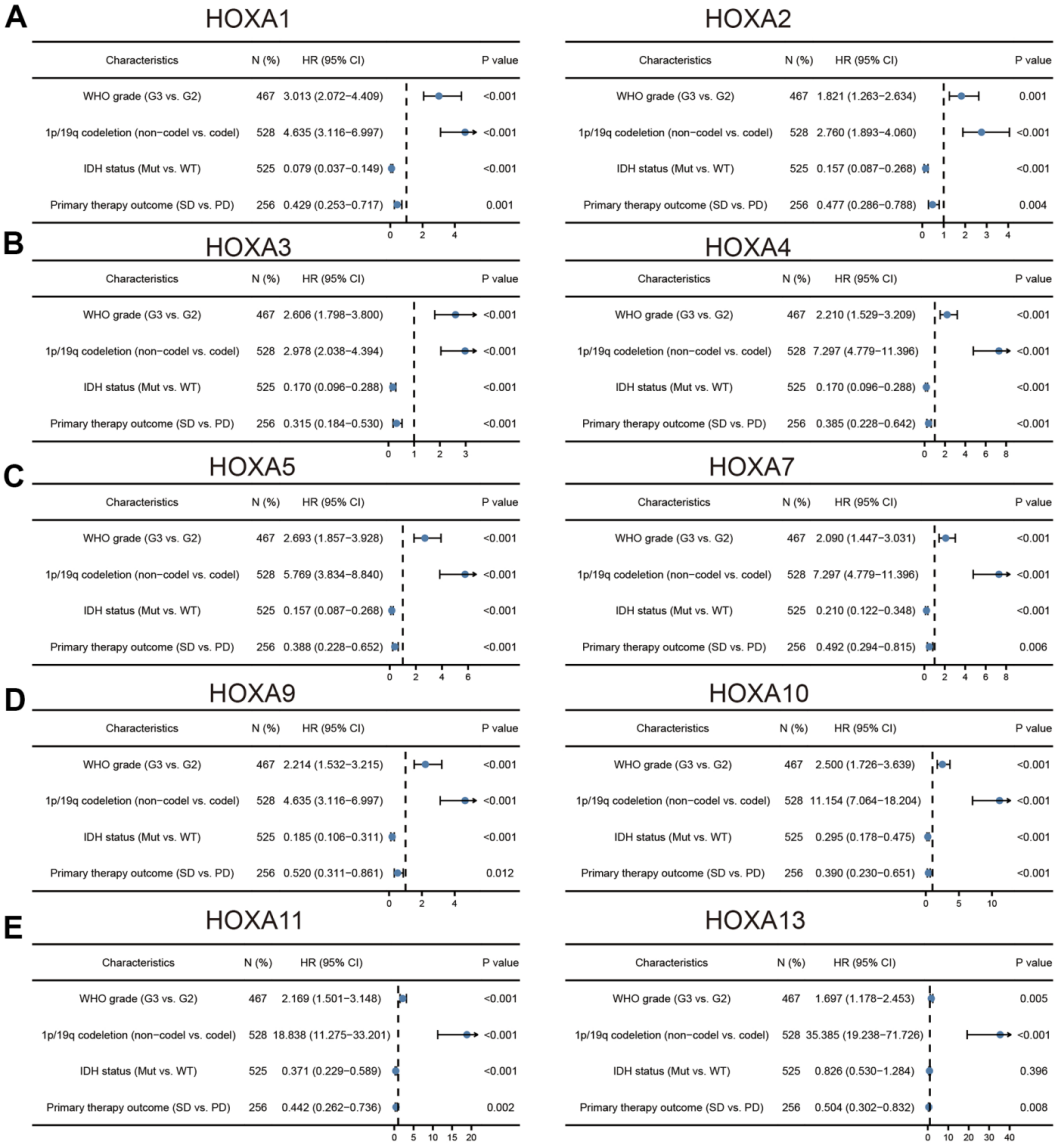
**Logistic regression analysis of correlation between clinic-pathological features and HOXAs expression in LGG patients.** (**A**–**E**) Logistic regression analysis of correlation between clinic-pathological features and HOXAs expression in LGG patients.

**Figure 5:**
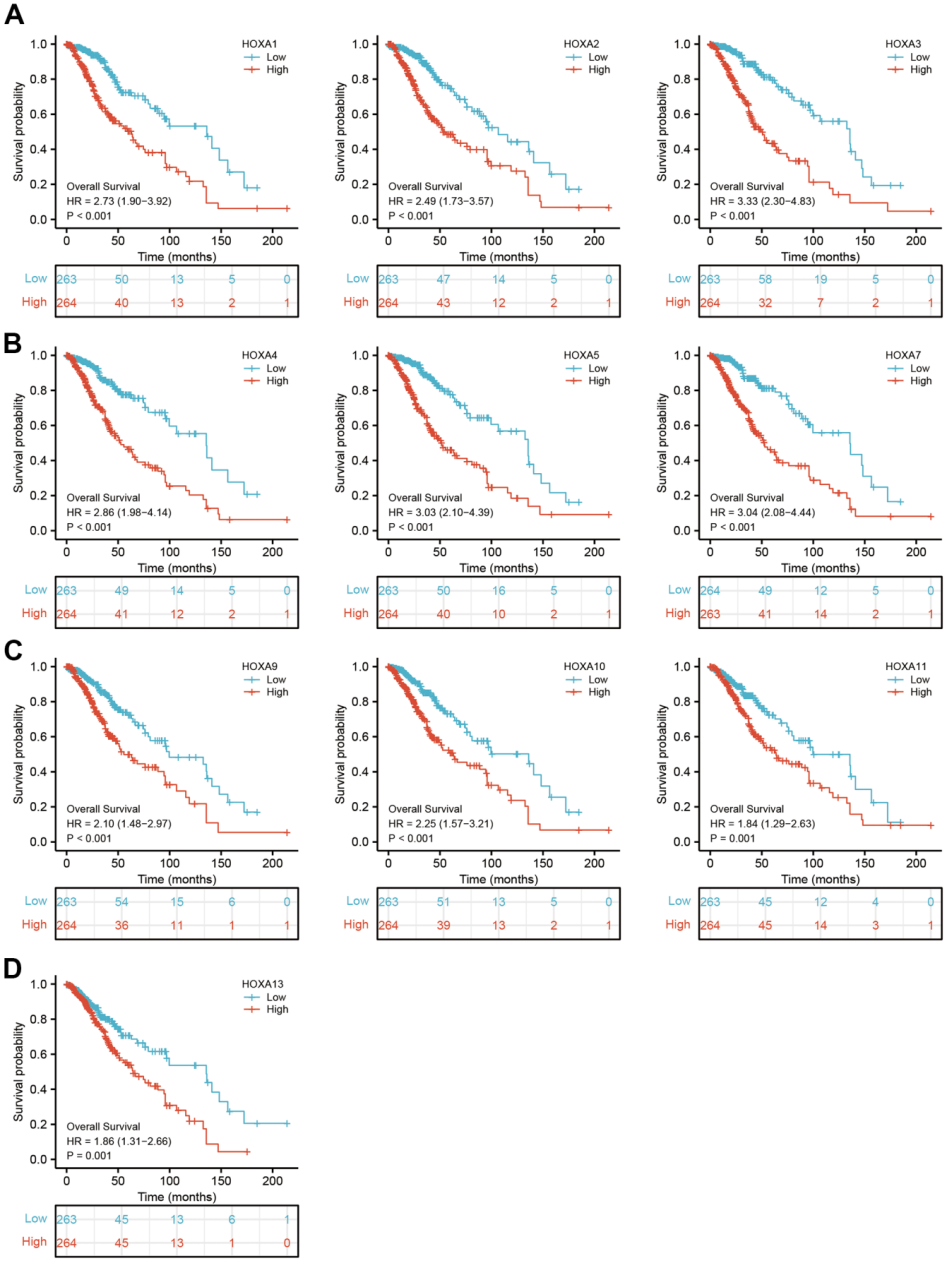
**The overall survival of HOXAs in LGG.** (**A**–**D**) The overall survival of HOXAs in LGG examine by TCGA database.

**Figure 6:**
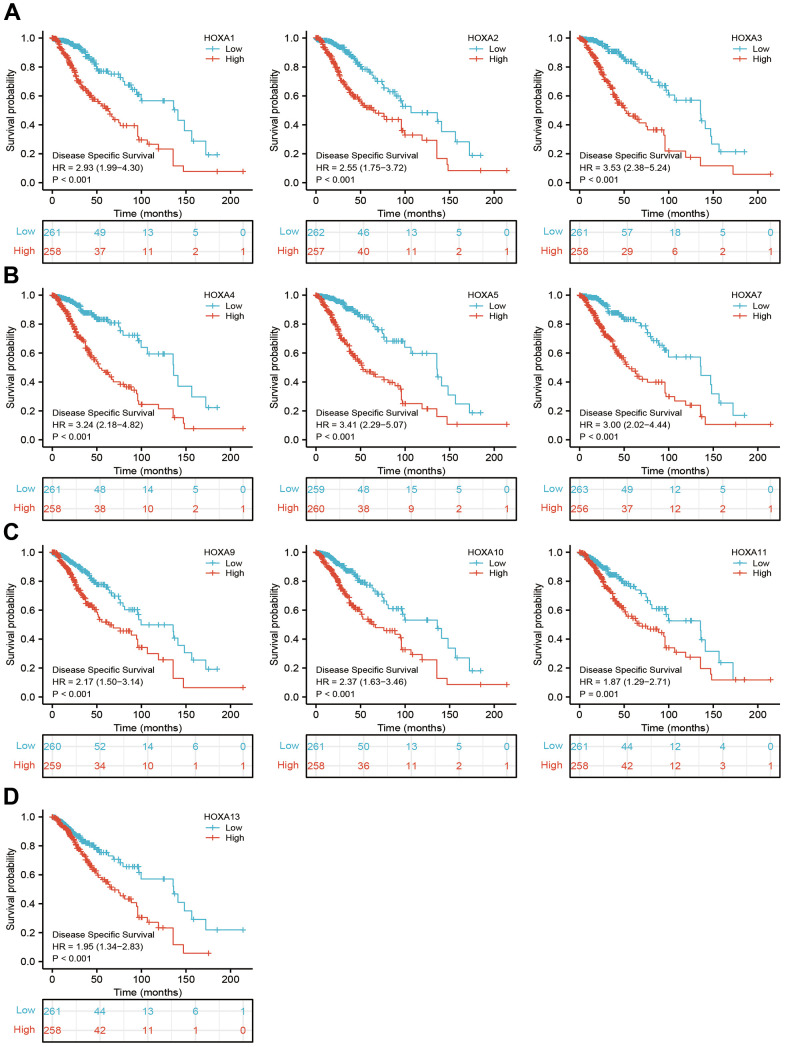
**The disease specific survival of HOXAs in LGG.** (**A**–**D**) The disease specific survival of HOXAs in LGG examine by TCGA database.

**Figure 7:**
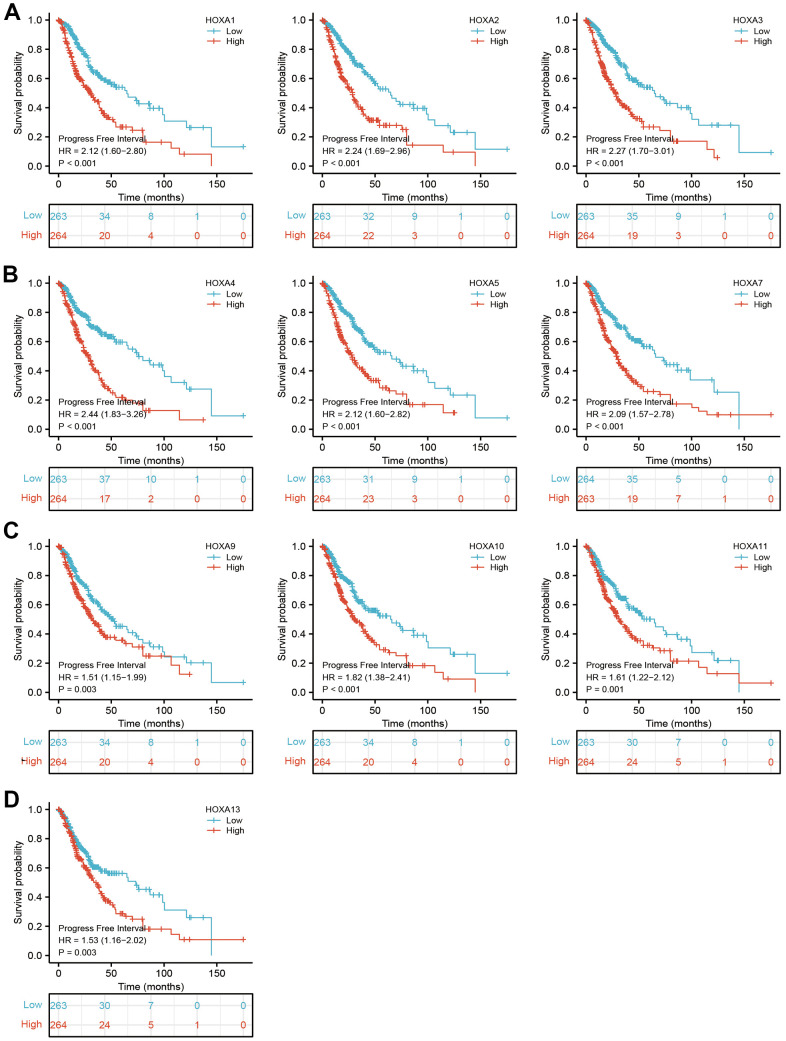
**The progression free survival of HOXAs in LGG.** (**A**–**D**) The progression free survival of HOXAs in LGG examine by TCGA database.

We further explored the correlation between HOXAs and clinical characteristics of LGG. The results showed that up-regulated 11 HOXAs were significantly associated with a higher tumor stage, IDH mutation status, 1p/19q chromosome co-deletion, histological type, primary therapy outcome, and age based on TCGA dataset (Figures 2, 3 and Supplementary Figure 1, Supplementary Table 1). This result was verified by CGGA database (Supplementary Figures 2, 3). The Logistic regression analysis of results also confirmed that HOXAs expression was remarkably associated with poor clinicopathological characteristics (Figure 4). Given that HOXAs were up-regulated in LGG and its higher expression associated with poor clinical characteristics. Therefore, we further explored the correlation among distinct HOXAs and the survival rate of LGG patients. The results indicated that higher expression of 11 HOXA was correlated with shorter overall survival (OS), disease-specific survival (DSS) and progression-free survival (PFS) in LGG patients (Figures 5–7). These results were verified by CGGA datasets (Supplementary Figure 4). Given that HOXAs differentially expression and associated with poor clinic-pathologic features and prognosis in LGG. The univariate cox proportional hazards regression analyses results confirmed that HOXA members (HOXA1, HOXA2, HOXA3, HOXA4, HOXA5, HOXA6, HOXA7, HOXA9, HOXA10 and HOXA11) expression and six clinical features (WHO grade, 1p/19q co-deletion, primary therapy outcome, IDH status, histological type and age) were correlated with adverse clinical outcomes of LGG patients. Additionally, the multivariate analyses revealed that HOXA1, HOXA6 expression and tumor grade, age, primary therapy outcome and age were independent factors affecting the prognosis of LGG patients (Table 1).

**Table 1:** Cox regression analysis for clinical outcomes in LGG patients.

**Characteristics**	**Total(N)**	**Univariate analysis**			**Multivariate analysis**	
		**Hazard ratio (95% CI)**	**P value**		**Hazard ratio (95% CI)**	**P value**
WHO grade	466					
G2	223					
G3	243	3.059 (2.046-4.573)	**<0.001**		3.041 (1.602-5.770)	**<0.001**
1p/19q codeletion	527					
codel	170					
non-codel	357	2.493 (1.590-3.910)	**<0.001**		0.838 (0.361-1.943)	0.680
Primary therapy outcome	457					
PD	110					
SD	146	0.439 (0.292-0.661)	**<0.001**		0.478 (0.251-0.908)	**0.024**
PR	64	0.175 (0.076-0.402)	**<0.001**		0.194 (0.058-0.654)	**0.008**
CR	137	0.122 (0.056-0.266)	**<0.001**		0.209 (0.083-0.524)	**<0.001**
IDH status	524					
WT	97					
Mut	427	0.186 (0.130-0.265)	**<0.001**		0.724 (0.268-1.959)	0.525
Histological type	393					
Astrocytoma	195					
Oligodendroglioma	198	0.577 (0.392-0.849)	**0.005**		0.925 (0.459-1.863)	0.826
Gender	527					
Female	238					
Male	289	1.124 (0.800-1.580)	0.499			
Race	508					
Black or African American	22					
White	486	0.686 (0.319-1.471)	0.333			
Age	527					
<=40	264					
>40	263	2.889 (2.009-4.155)	**<0.001**		3.862 (2.045-7.293)	**<0.001**
HOXA1	527	2.049 (1.778-2.362)	**<0.001**		2.603 (1.316-5.147)	**0.006**
HOXA2	527	1.582 (1.419-1.763)	**<0.001**		1.116 (0.657-1.896)	0.685
HOXA3	527	1.516 (1.367-1.682)	**<0.001**		0.653 (0.320-1.333)	0.242
HOXA4	527	1.611 (1.454-1.785)	**<0.001**		1.132 (0.677-1.890)	0.637
HOXA5	527	1.442 (1.334-1.559)	**<0.001**		1.353 (0.718-2.549)	0.350
HOXA6	527	1.546 (1.385-1.726)	**<0.001**		0.409 (0.220-0.758)	**0.005**
HOXA7	527	1.615 (1.448-1.801)	**<0.001**		1.590 (0.886-2.852)	0.120
HOXA9	527	1.345 (1.210-1.494)	**<0.001**		0.881 (0.529-1.465)	0.625
HOXA10	527	1.350 (1.242-1.467)	**<0.001**		0.962 (0.593-1.561)	0.875
HOXA11	527	1.295 (1.168-1.436)	**<0.001**		1.262 (0.711-2.240)	0.427
HOXA13	527	1.131 (0.994-1.286)	0.062		0.991 (0.643-1.526)	0.966

### Diagnostic values of HOXAs in LGG

**Figure 8:**
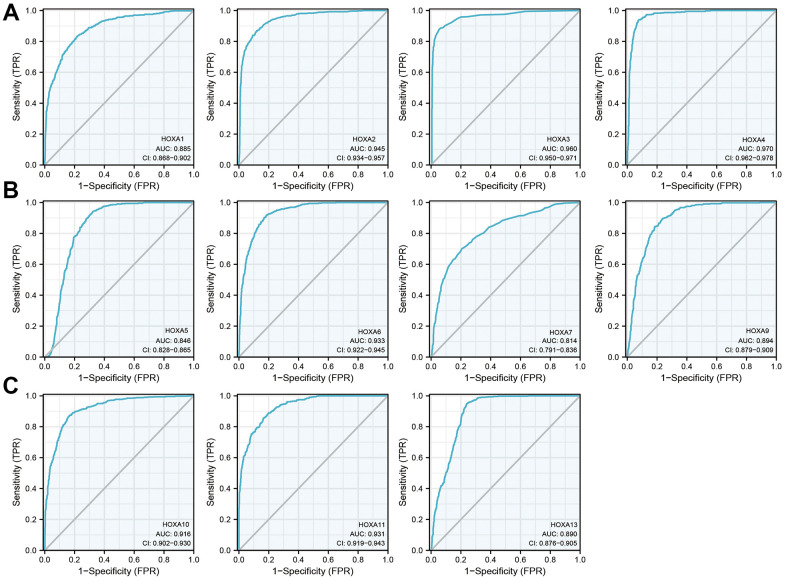
**The diagnosis values of HOXAs in LGG.** (**A**–**C**) ROC curve analysis the diagnosis values of HOXAs in LGG.

Given that HOXAs were significantly up-regulated in LGG and its higher expression was associated with adverse clinical features and adverse clinical outcomes. Therefore, we decided explored the diagnosis values of HOXAs in LGG. ROC curve analysis of HOXAs showed that HOXAs had a high accuracy (AUC > 0.80) in predicting LGG (Figure 8). These results confirmed that HOXAs has the potential to act as a diagnosis biomarker for the diagnosis of LGG with high sensitivity and specificity.

### Construction and validation of HOXAs based nomogram

**Figure 9:**
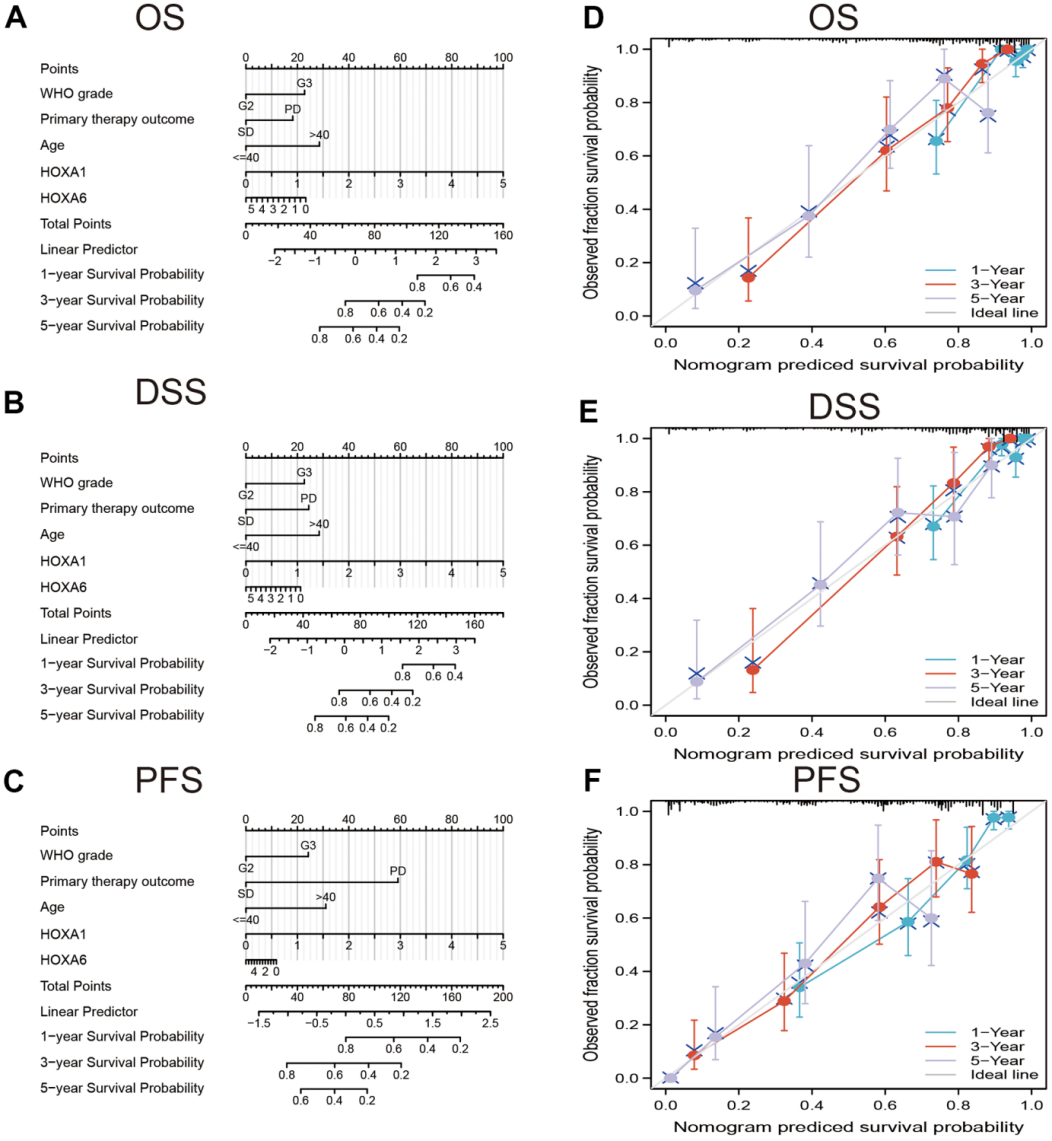
**Construction and performance validation of the HOXAs based nomogram for LGG patients.** Nomogram to predict (**A**) OS, (**B**) DSS and (**C**) PFI for lung cancer patients. The calibration curve and Hosmer–Lemeshow test of nomograms in the TCGA-LGG cohort for (**D**) OS, (**E**) DSS, and (**F**) PFI.

In order to provide clinicians with predicted prognosis of LGG patients quantitatively, we constructed the nomogram combining HOXA1, HOXA6 expression and independent clinical risk factors (tumor grade, age, primary therapy outcome and age). Higher total points on the nomogram for overall survival (OS), disease-specific survival (DSS) and progression-free survival (PFS), respectively, indicated a worse prognosis (Figure 9). In summary, these results indicate that the nomogram can well predict short- or long-term survival of LGG patients.

### Gene mutation analysis

**Figure 10:**
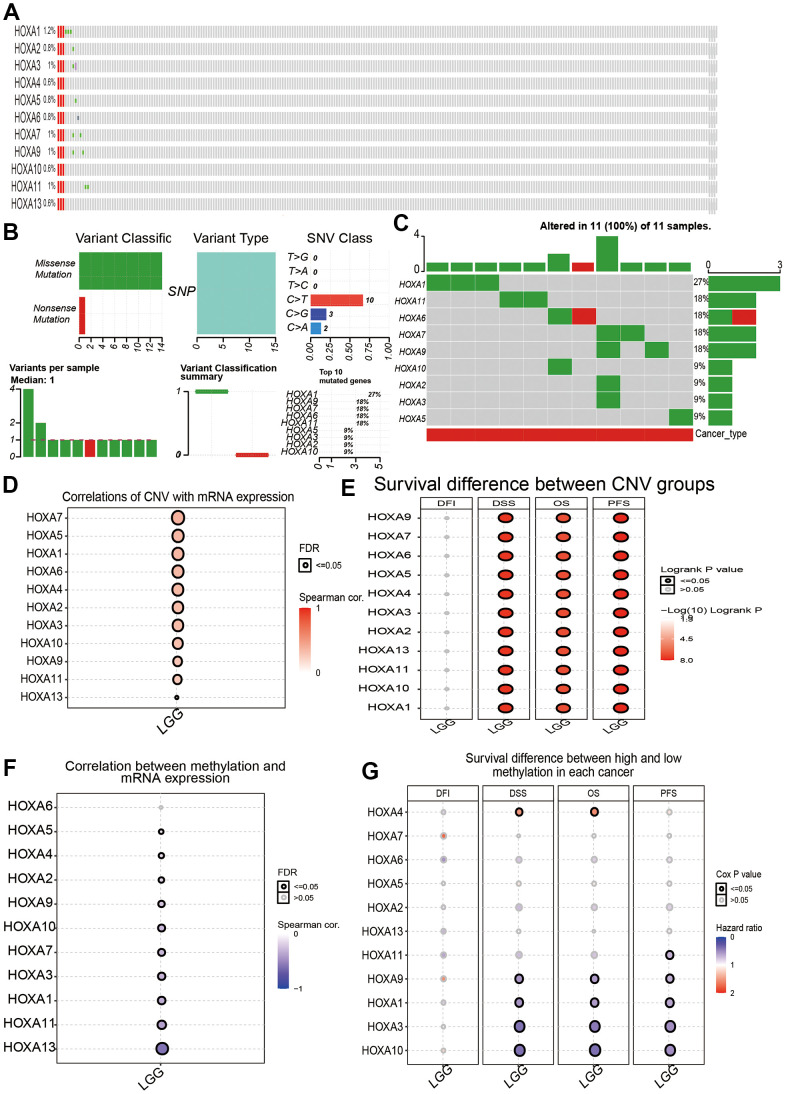
**Landscape of genetic and expression variation of HOXAs in LGG.** (**A**–**C**) Analysis gene alteration of HOXA gene family in LGG by used cBioPortal database. (**D**) Analysis the correlation between CNV and its expression. (**E**) Analysis prognosis of CNV for HOXAs in LGG. (**F**) Analysis the correlation between DNA methylation and its expression. (**G**) Analysis prognosis of DNA methylation for HOXAs in LGG.

We utilized cBioPortal analysis the genetic alteration of differentially expressed HOXAs family. Results confirmed that HOXA1, HOXA2, HOXA3, HOXA4, HOXA5, HOXA6, HOXA7, HOXA9, HOXA10, HOXA11 and HOXA13 were all altered, with 1.2, 0.8, 1, 0.6, 0.8, 0.8, 1, 1, 0.6, 1 and 0.6alterations in the LGG samples, respectively (Figure 10A). We also summarize the incidence of CNV and somatic mutations of 11 HOXAs in LGG. The results confirmed that missense mutation was the most common variant classification, SNPs were the most common variant type and C > T ranked as the top SNV class. The results also showed HOXA1 as the gene with the highest mutation frequency, followed by HOXA11 and HOXA6, among the 11 HOXAs (Figure 10B, 10C). CNV analysis results confirmed that copy number variations of HOXAs was significantly positive correlated with its expression in LGG (Figure 10D). Furthermore, HOXAs CNV affect the overall survival (OS), disease-specific survival (DSS) and progression-free survival (PFS) in LGG patients (Figure 10E). Finally, we uncover that the DNA methylation level of HOXA13 negatively associated with its expression (Figure 10F). More importantly, lower DNA methylation level of HOXA1, HOXA3, HOXA9 and HOXA10 were correlated with poor DSS, OS and PFS (Figure 10G).

### Functional analysis of HOXAs in LGG

**Figure 11:**
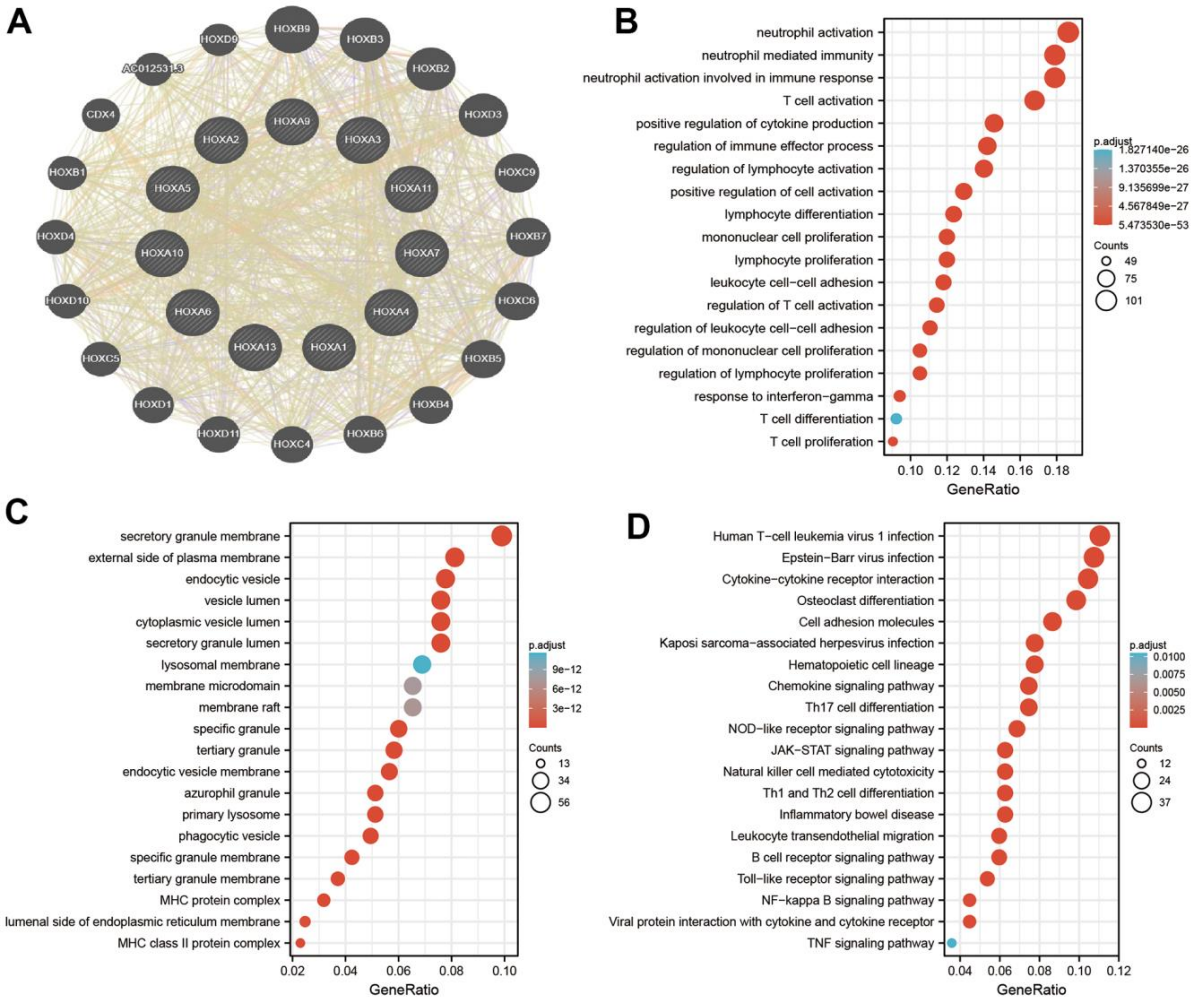
**Analysis the function of HOXAs in LGG.** (**A**) Analysis the co-expression genes of HOXAs in LGG examined by GeneMANIA databases. (**B, C**) Analysis the GO term of HOXAs in LGG. (**D**) Analysis the KEGG signaling pathway of HOXAs in LGG.

To explore the potential functions of HOXAs in LGG progression, we were using the GeneMANIA database constructed the gene-gene interaction network for HOXAs and the altered neighboring genes (Figure 11A). Furthermore, we perform the GO and KEGG enrichment to explore the functions of HOXAs and their neighboring genes in LGG. The enrichment results confirmed that the biological processes for these genes were mostly neutrophil activation, neutrophil mediated immunity, neutrophil activation involved in immune response, T cell activation, positive regulation of cytokine production, regulation of immune effector process, regulation of lymphocyte activation, positive regulation of cell activation, lymphocyte differentiation, mononuclear cell proliferation, lymphocyte proliferation, leukocyte cell−cell adhesion, regulation of T cell activation, regulation of leukocyte cell−cell adhesion, regulation of mononuclear cell proliferation, regulation of lymphocyte proliferation, response to interferon−gamma, T cell differentiation and T cell proliferation (Figure 11B). The molecular functions for these genes were mostly secretory granule membrane, external side of plasma membrane, lysosomal membrane, membrane microdomain, phagocytic vesicle, specific granule membrane, MHC protein complex and MHC class II protein complex (Figure 11C). KEGG enrichment results confirmed that these genes mainly involved in the Cytokine−cytokine receptor interaction, Osteoclast differentiation, Cell adhesion molecules, Kaposi sarcoma−associated herpes, virus infection, Hematopoietic cell lineage, Chemokine signaling pathway, Th17 cell differentiation, NOD−like receptor signaling pathway, JAK−STAT signaling pathway, Natural killer cell mediated cytotoxicity, Th1 and Th2 cell differentiation, Inflammatory bowel disease, Leukocyte transendothelial migration, B cell receptor signaling pathway, Toll−like receptor signaling pathway, NF−kappa B signaling pathway, Viral protein interaction with cytokine and cytokine receptor and TNF signaling pathway (Figure 11D). Collectively, these data suggest that HOXAs plays an essential regulatory role in the inflammation response and immune regulation.

### Correlation between HOXAs and immune subtypes of LGG

For determine the expression of HOXAs in diverse immune subtypes of LGG, we utilized the TISIDB database analysis and found that HOXAs mainly highly expressed in C3 and C4 subtype (Supplementary Figure 5).

### Immune infiltration analysis of the HOXAs in LGG

**Figure 12:**
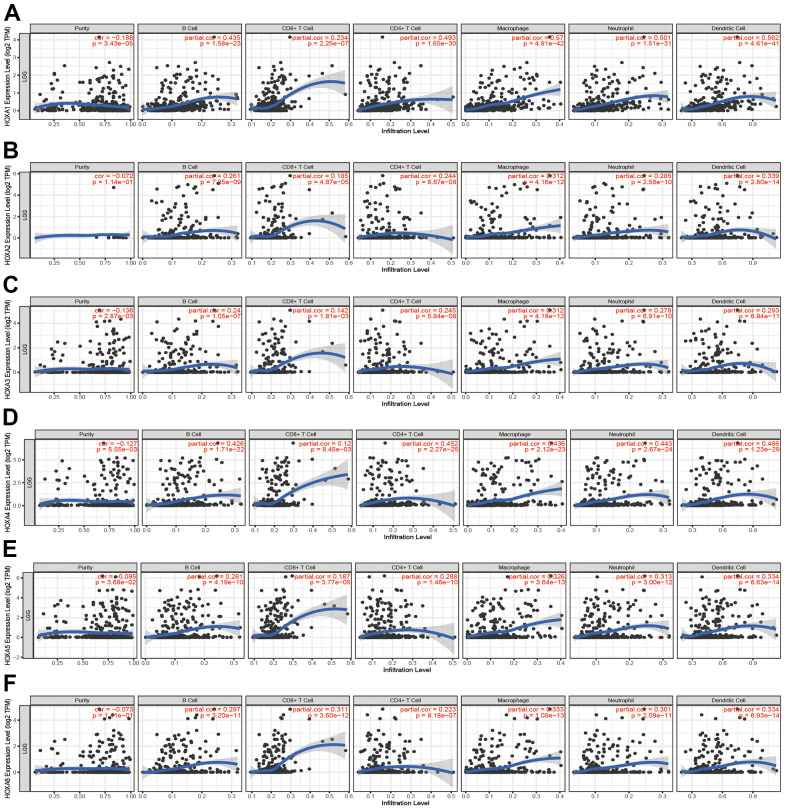
**Analysis the correlation between HOXAs expression and diverse immune cell infiltration.** (**A**–**F**) The correlation between HOXAs expression and diverse immune cell infiltration in LGG examine by TIMER database. *p < 0.05, **p < 0.01, ***p < 0.001.

Given that HOXAs was significantly correlated with immune regulation related signaling pathway. Therefore, we decide to examine the correlation between HOXAs expression and immune cell infiltration. In this finding, we used TIMER database analysis and found that somatic copy number alterations of HOXAs were significantly correlated with diverse immune cell infiltration levels in LGG (Supplementary Figures 6, 7). Furthermore, we also utilized the TIMER database analysis the correlation between HOXAs and diverse immune cell. The results confirm that HOXAs were positively correlated with the immune infiltration of B cells, CD4+ T cells, CD8+ T cells, Neutrophils, Macrophages and Dendritic cells in LGG (Figure 12 and Supplementary Figure 8).

**Figure 13:**
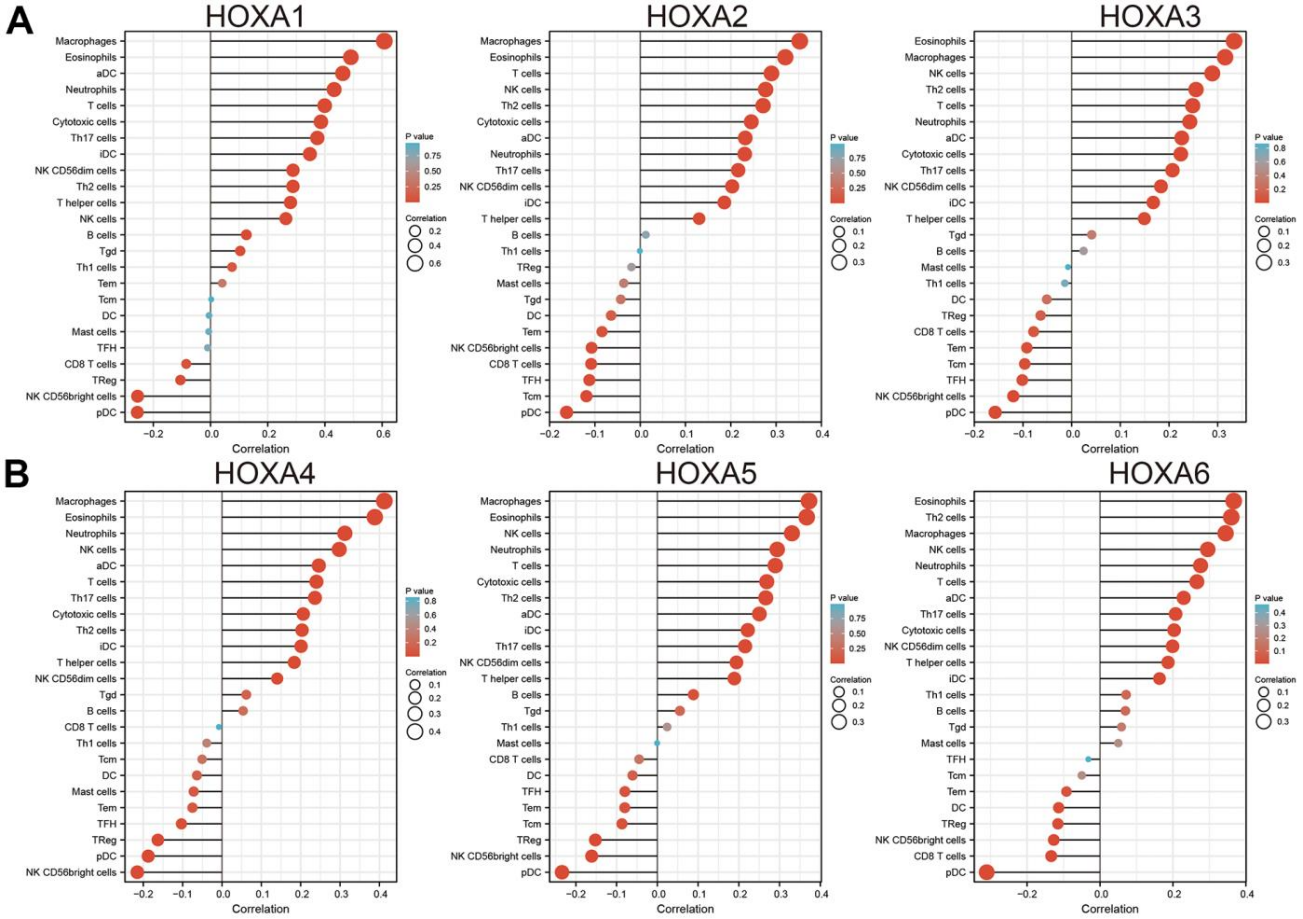
**Analysis the correlation between HOXAs expression and diverse immune cell infiltration.** (**A**, **B**) Analysis correlation between HOXAs expression and diverse immune cell infiltration examine by GSVA R package. *p < 0.05, **p < 0.01, ***p < 0.001.

Finally, ssGSEA with Spearman’s rank correlation was employed to measure the correlation between HOXAs expression and infiltration levels of 24 immune cell types in glioma. The results showed that HOXA1 expression was correlated with the immune infiltration of macrophages and eosinophils, negatively correlated with the immune infiltration of NK CD56bright cells and pDC. HOXA2 positively correlated with the immune infiltration of Macrophages, Eosinophils and T cells, negatively correlated with the immune infiltration of Tcm and pDC. HOXA3 positively correlated with the immune infiltration of NK cells, Th2 cells and T cells, negatively correlated with the immune infiltration of NK CD56bright cells and pDC. HOXA4 positively correlated with the immune infiltration of macrophages, eosinophils and neutrophils, negatively correlated with the immune infiltration of pDC and NK CD56bright cells. HOXA5 positively correlated with the immune infiltration of Macrophages, Eosinophils, NK cells and Neutrophils, negatively correlated with the immune infiltration of NK CD56bright cells and pDC. HOXA6 positively correlated with the immune infiltration of Eosinophils, Th2 cells and Macrophages, negatively correlated with the immune infiltration of CD8 T cells and pDC. HOXA7 positively correlated with the immune infiltration of Th2 cells, neutrophils and NK cells, negatively correlated with the immune infiltration of NK CD56bright cells and pDC. HOXA9 positively correlated with the immune infiltration of Neutrophils and Eosinophils, negatively correlated with the immune infiltration of NK CD56bright cells and TReg. Furthermore, HOXA10 expression associated with the immune infiltration of several subsets of myeloid cells, including macrophages, Eosinophils and Neutrophils, HOXA10 expression negatively correlated with the immune infiltration of TReg and NK CD56bright cells. There are positive correlations between HOXA11 expression and the immune infiltration of Macrophages, T helper cells and Neutrophils, negatively correlated with the immune infiltration of TRegNK CD56bright cells. There is also a strong positive correlation between HOXA13 expression and immune infiltration of Tgd, T helper cells and Macrophages, negatively correlated with the immune infiltration of TReg and NK CD56bright cells (Figure 13 and Supplementary Figure 9). Taken together, these results confirmed that HOXAs plays a pivotal role in the immune regulation.

### Correlation between HOXAs and immune modulator in LGG

**Figure 14:**
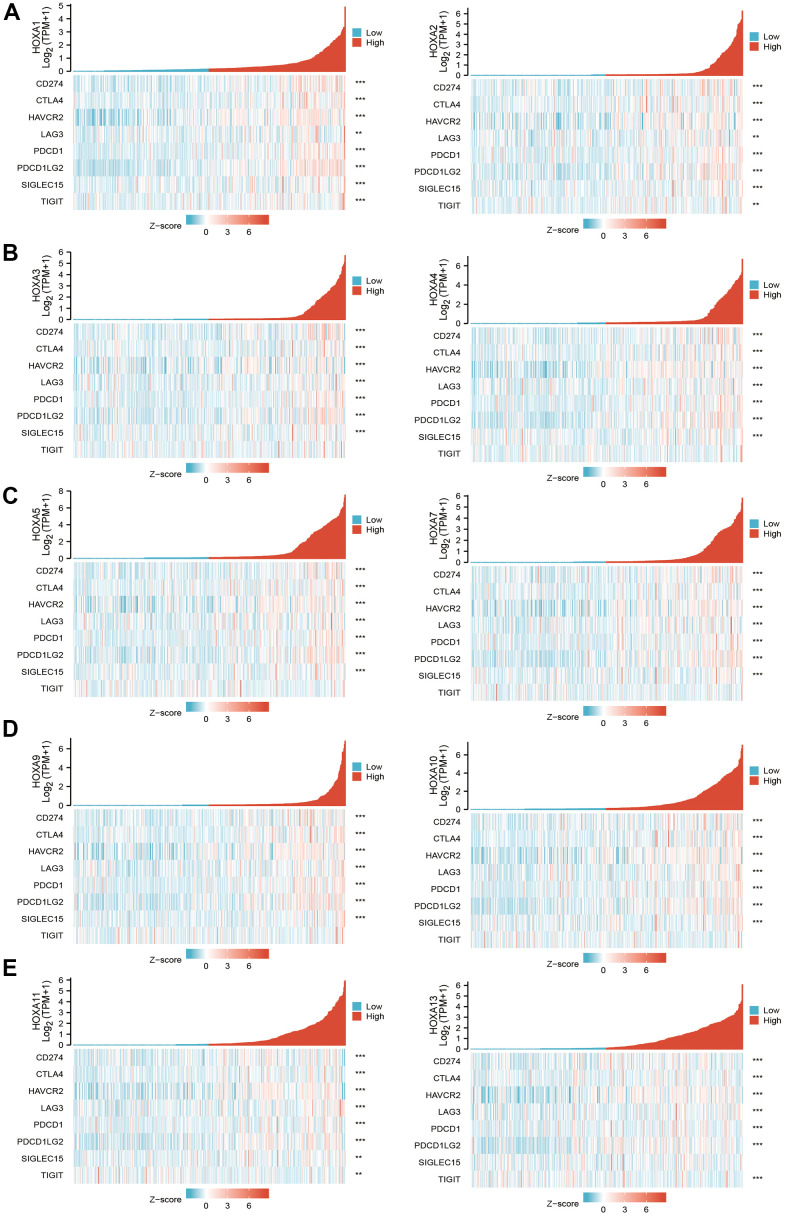
**The correlation between HOXAs expression and immune modulator.** (**A**–**E**) The correlation between HOXAs expression and immune modulator.

Considering immune modulator plays a crucial role in the immune response and development and progression of LGG. We explored the correlation between HOXAs and the immune checkpoint-related genes by using Pearson correlation analysis in LGG, including CD274, CTLA4, HAVCR2, LAG3, PDCD1, PDCD1LG12, TIGIT, SIGLEC15. The result showed that HOXAs expression was significantly positively correlated with CD274, CTLA4, HAVCR2, LAG3, PDCD1, PDCD1LG12 and SIGLEC15 (Figure 14). These results demonstrate that HOXAs expression was significantly correlated with the expression of immune checkpoint-related genes in LGG.

### Correlation between HOXAs expression and diverse drug sensitivity

We downloaded the IC50 values of anti-cancer drugs and gene expression profiles in the relative cell lines from the GDSC and CTRP database. To explore the influence of HOXAs expression on the sensitivity of anti-cancer drugs, we divided the tumor cells into high- and low- HOXAs groups and compared their IC50 values. For example, we found that the IC50 values of several anti-cancer drugs decreased in the high- HOXA3 group, including abiraterone, Trametinib, VAF-347, BRD-K09344309, BRD-K34099515 and erlotinib (Supplementary Figure 10), indicating that patients exhibiting high HOXA3 expression levels are relatively sensitive to these anti-cancer drugs. We also uncover that HOXAs were positively correlated with the diverse drug sensitivity. For instance, HOXA3 was positively correlated with the diverse drug sensitivity of tacedinaline, BRD-A94377914, LRRK2-IN-1, PX-12, Apicidin, PRIMA-1, Necrosulfonamide, tipifarnib-P2, vorinostat, avrainvillamide, serdemetan, cerulenin, CCT036477 and PL-DI (Supplementary Figure 10). Taken together, these results confirmed that HOXAs was positively or negatively correlated with the diverse drug sensitivity in the cancer therapeutic response portal database.

### Construction of a network of HOXAs related ceRNA

Emerging reports have revealed that lncRNA /miRNA regulatory axis play a vital role in the gene expression [25]. To uncover that the upstream regulatory mechanism of HOXAs in LGG, we utilized the LnCeVar database identification of HOXAs related ceRNA events in LGG of patients from TCGA. The results confirmed that ZHF337-AS1 or SNHG12/miR-210-3p / regulatory axis may regulate the expression of HOXA1. LINC00174/miR-26a-5p regulatory axis may regulate the expression of HOXA5. HCG18 or TRG- AS1/miR-196a-5p regulatory axis may regulate the expression of HOXA7. LNC00665/miR-320a regulatory axis may regulate he expression of HOXA10. KDM4A-AS1/ miR-27a-5p regulatory axis may regulate he expression of HOXA13 (Supplementary Figure 11). These results confirmed that HOXAs related ceRNA network regulatory axis may play a vital role in the progression of LGG.

### Knock down of HOXA6 inhibits the cell proliferation and migration of LGG cells

**Figure 15:**
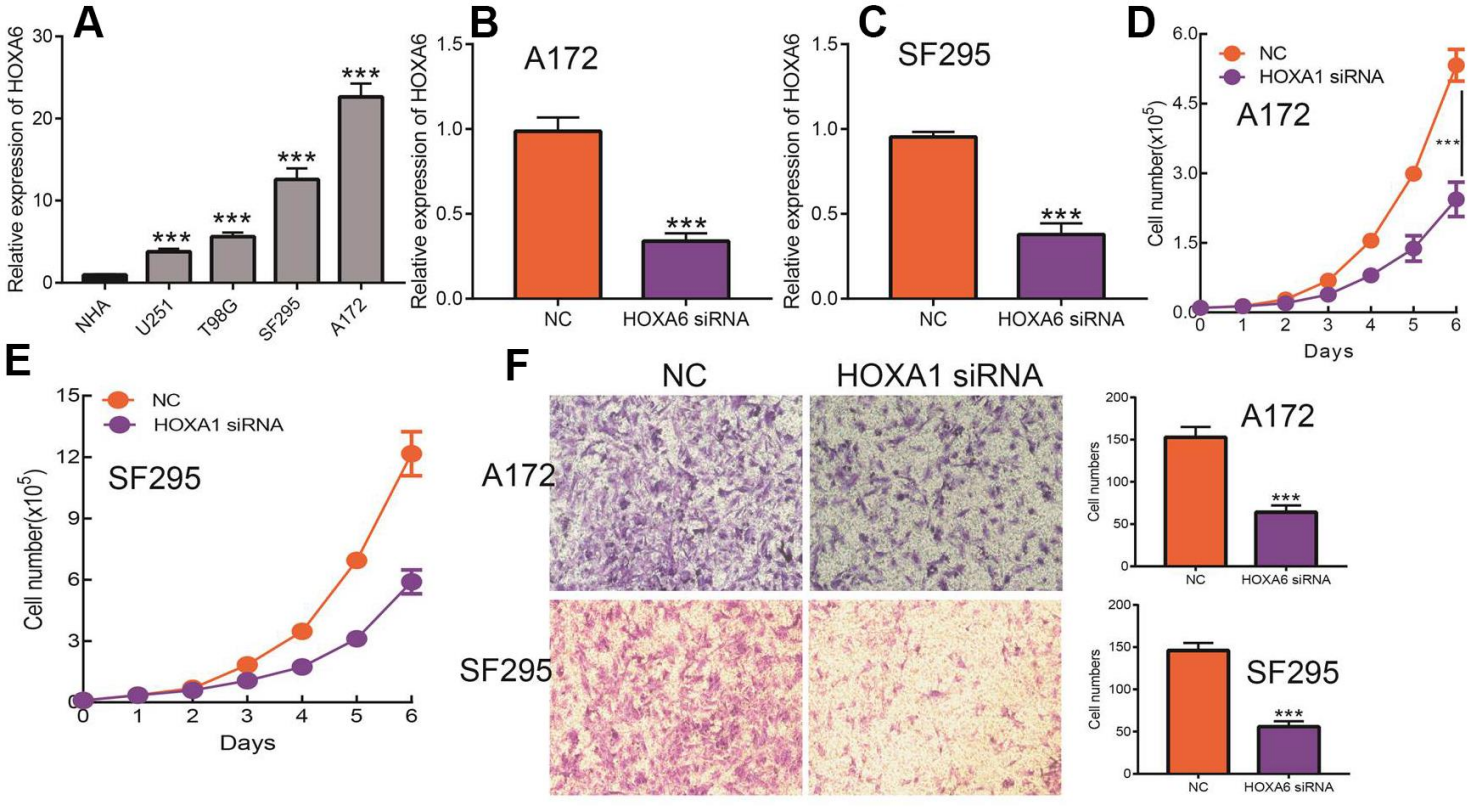
**Knock down of HOXA6 inhibits LGG cell proliferation and migration.** (**A**) The expression of HOXA6 in LGG cells lines examined by qRT-PCR assay. (**B**, **C**) The knock down efficiency of HOXA6 in LGG cells lines examined by qRT-PCR assay. (**D**, **E**) Depletion of HOXA6 inhibits LGG cells proliferation examined by growth curve assay. (**F**) Depletion of HOXA6 inhibits LGG cells migration examined by transwell assay.

Given that HOXA1 expression was independent factors affecting the prognosis of LGG patients and previous no study reported the function of HOXA6 in LGG. Therefore, we decide explored function of HOXA6 in LGG. We first examined the expression of HOXA6 in LGG cells lines by qRT-PCR assay. The results confirmed that HOXA6 was up-regulated in LGG cells lines, especially in SF295 and A172 cells (Figure 15A). Next, we utilized siRNA knock down of HOXA6 in SF295 and A172 cells, the knock down efficiency was verify by qRT-PCR assay (Figure 15B, 15C). The growth curve assay showed that depletion of HOXA6 significantly inhibits LGG cells proliferation ability (Figure 15D, 15E). Furthermore, the transwell assay confirmed that HOXA6 knock down significantly inhibits LGG cells migration ability (Figure 15F). Collectively, these results demonstrate that HOXA6 was highly expressed in LGG cells lines and significantly affected their proliferation and migration abilities.

## DISCUSSION

Grades I and II are grouped together as low-grade gliomas and grades III and IV as high-grade gliomas, low-grade gliomas have a 10- to 15-year survival. Although earlier diagnosis and newer therapies have increased overall survival, disparities in access and outcomes of care for low-grade gliomas persist [26]. Therefore, more sensitive and specific diagnostic biomarkers and potential therapeutic targets for this cancer type need to be identified.

Previous studies reported that HOXA genes differentially expressed in various cancers. For instance, it was found that HOXA7 and HOXA9 mRNAs were elevated in esophageal squamous cell carcinoma tissues [27]. On the contrary, HOXA9 was decreased in NSCLC [28]. HOXA13 expression was up-regulated in in breast cancer [28], whereas it was down-regulated in colorectal cancer [29]. However, the expression pattern of the HOXA gene family in LGG was not previously comprehensively investigated. In our finding, we found that 11 HOXAs were significantly higher in LGG and GBM than normal samples. Furthermore, we uncover that the protein of HOXA4, HOXA6, HOXA9 and HOXA10 were highly expressed in LGG tissue compared with control group based on the Human Protein Atlas datasets.

It has been showed that higher level of HOXA9 and HOXA10 were found to be predictors of poor outcome in patients with NSCLC and GBM [30]. Additionally, multiple highly expressed HOXA members were found to be significantly associated with adverse clinical outcomes in acute myeloid leukemia patients [31]. In this research, we uncover that up-regulated 11 HOXAs were significantly associated with a higher tumor stage, IDH mutation status, 1p/19q chromosome co-deletion, histological type and primary therapy outcome based on TCGA dataset. These results were verified by CGGA. The cox regression analyses result also confirmed that HOXAs expression were remarkably associated with poor clinicopathological characteristics. Given that HOXAs were up-regulated in LGG and its higher expression associated with poor clinical characteristics. Therefore, we further explored the correlation among distinct HOXAs and the survival rate of LGG patients. The results indicated that higher expression of 11 HOXA was correlated with shorter overall survival (OS), disease-specific survival (DSS) and progression-free survival (PFS) in LGG patients. These results were verified by CGGA datasets. Given that HOXAs differentially expression and associated with poor clinic-pathologic features and prognosis in LGG. The univariate cox proportional hazards regression analyses results confirmed that HOXA members (HOXA1, HOXA2, HOXA3, HOXA4, HOXA5, HOXA6, HOXA7, HOXA9, HOXA10 and HOXA11) expression and six clinical features (WHO grade, 1p/19q co-deletion, primary therapy outcome, IDH status, histological type and age) were correlated with adverse clinical outcomes of LGG patients. Additionally, the multivariate analyses revealed that HOXA1, HOXA6 expression and tumor grade, age, primary therapy outcome and age were independent factors affecting the prognosis of LGG patients. Zhou et al. found that HOXA10 may be as a potential prognosis biomarker for the prediction of poor outcome in LSCC patients. In this study, we found that HOXAs showed that HOXAs had a high accuracy (AUC > 0.80) in predicting LGG patients.

We also analysis the landscape of genetic variation of HOXAs in LGG, the results confirmed that HOXA1, HOXA2, HOXA3, HOXA4, HOXA5, HOXA6, HOXA7, HOXA9, HOXA10, HOXA11 and HOXA13 were all altered, with 1.2, 0.8, 1, 0.6, 0.8, 0.8, 1, 1, 0.6, 1 and 0.6alterations in the LGG samples, respectively. We also uncover that missense mutation was the most common variant classification, SNPs were the most common variant type and C > T ranked as the top SNV class. The results also showed HOXA1 as the gene with the highest mutation frequency, followed by HOXA11 and HOXA6, among the 11 HOXAs. CNV analysis results confirmed that copy number variations of HOXAs was significantly positive correlated with its expression in LGG. Furthermore, HOXAs CNV affect the overall survival (OS), disease-specific survival (DSS) and progression-free survival (PFS) in LGG patients. Finally, we uncover that the DNA methylation level of HOXA13 negatively associated with its expression. More importantly, lower DNA methylation level of HOXA1, HOXA3, HOXA9 and HOXA10 were correlated with poor DSS, OS and PFS.

Past studies revealed that HOXAs played a key role in the regulating cell differentiation, proliferation, migration and cell death. For example, it has been found that HOXA10 was highly expressed in hepatocellular carcinoma. Depletion of HOXA10 induced cell cycle arrest at the G0/G1 phase and apoptosis by decreased the expression of CCND1 and survivin [32]. On the other hand, studies showed that HOXA11 was significantly lower in cisplatin-resistant lung adenocarcinoma cell line. Elevated HOXA11 expression was significantly improved the cisplatin sensitivity by inhibiting Akt/β-catenin signaling [33]. In the present study, we uncover that HOXAs genes mainly involve in the biology process, including the neutrophil activation, neutrophil mediated immunity, neutrophil activation involved in immune response, T cell activation, positive regulation of cytokine production, regulation of immune effector process, regulation of lymphocyte activation, positive regulation of cell activation, lymphocyte differentiation, mononuclear cell proliferation, lymphocyte proliferation, leukocyte cell−cell adhesion, regulation of T cell activation, regulation of leukocyte cell−cell adhesion, regulation of mononuclear cell proliferation, regulation of lymphocyte proliferation, response to interferon−gamma, T cell differentiation and T cell proliferation. Furthermore, we demonstrated that HOXAs genes mainly participated in the signaling pathway, including the Cell adhesion molecules, Kaposi sarcoma−associated herpes, virus infection, Hematopoietic cell lineage, Chemokine signaling pathway, Th17 cell differentiation, NOD−like receptor signaling pathway, JAK−STAT signaling pathway, Natural killer cell mediated cytotoxicity, Th1 and Th2 cell differentiation, Inflammatory bowel disease, Leukocyte transendothelial migration, B cell receptor signaling pathway, Toll−like receptor signaling pathway, NF−kappa B signaling pathway, Viral protein interaction with cytokine and cytokine receptor and TNF signaling pathway. Collectively, these data suggest that HOXAs plays an essential regulatory role in the inflammation response and immune regulation. Therefore, targeting HOXAs may be as attractive and alternative strategies for immunotherapy.

Previous studies showed that the immune infiltration of CD4+ T cells and CD8+ T cells were significantly affected the prognosis of LGG [34]. In this finding, we found that somatic copy number alterations of HOXAs were significantly correlated with diverse immune cell infiltration levels in LGG. Furthermore, we confirm that HOXAs were positively correlated with the immune infiltration of B cells, CD4+ T cells, CD8+ T cells, Neutrophils, Macrophages and Dendritic cells in LGG. More importantly, we uncover that HOXAs expression was significantly positively correlated with CD274, CTLA4, HAVCR2, LAG3, PDCD1, PDCD1LG12 and SIGLEC15. Thus, this research provides new insights into understanding the potential roles of HOXAs in immune cell infiltration of LGG and its potential use as cancer therapeutic and prognostic biomarker.

Finally, we also constructed HOXAs related mRNA–miRNA–lncRNA network in LGG. The results confirmed that ZHF337-AS1 or SNHG12/miR-210-3p / regulatory axis may regulate the expression of HOXA1. LINC00174/miR-26a-5p regulatory axis may regulate the expression of HOXA5. HCG18 or G18 or TRG-AS1/miR-196a-5p regulatory axis may regulate he expression of HOXA7. LNC00665/miR-320a regulatory axis may regulate he expression of HOXA10. KDM4A-AS1/ miR-27a-5p regulatory axis may regulate he expression of HOXA13. Previous study found that lncRNA SNHG12 promotes the cell proliferation, invasion and epithelial-mesenchymal transition of pancreatic cancer cells by absorbing miRNA-320b [35]. Li et al. found that lncRNA TRG-AS1 facilitate NSCLC cell proliferation and invasion by the miR-224-5p/SMAD4 axis [36].

Jiang et al. found that HOXA6 was over-expressed in in CRC tumor tissue than that in adjacent normal tissue. Depletion of HOXA6 inhibits the cell proliferation, migration and invasion, but inhibited apoptosis [37]. Xin et al. reported that HOXA6 by suppressing the PI3K/Akt signaling pathway and inhibits the cell proliferation and induces apoptosis in clear cell renal cell carcinoma [38]. However, there is not any study reported the function of HOXA6 in LGG. Therefore, we decide explored function of HOXA6 in LGG. We uncover that HOXA6 was up-regulated in LGG cells lines, especially in SF295 and A172 cells. Depletion of HOXA6 significantly inhibits LGG cells proliferation and migration abilities.

## CONCLUSIONS

HOXAs participated in the progression of LGG and were differentially expressed in LGG tissues. HOXA6 may be potential biomarker for the diagnosis of LGG.

## Supplementary Material

Supplementary Figures

Supplementary Table 1
