# Supplementary material for: Next-generation sequencing identifies HOXA6 as a novel oncogenic gene in low grade glioma
Source: Aging (Albany NY). 2022 Mar 29;14(6):2819–54. doi: 10.18632/aging.203977 (PMC9004573; doi:10.18632/aging.203977)
Supplement: Supplementary Figures [file aging-14-203977-s001.pdf]

SUPPLEMENTARY FIGURES

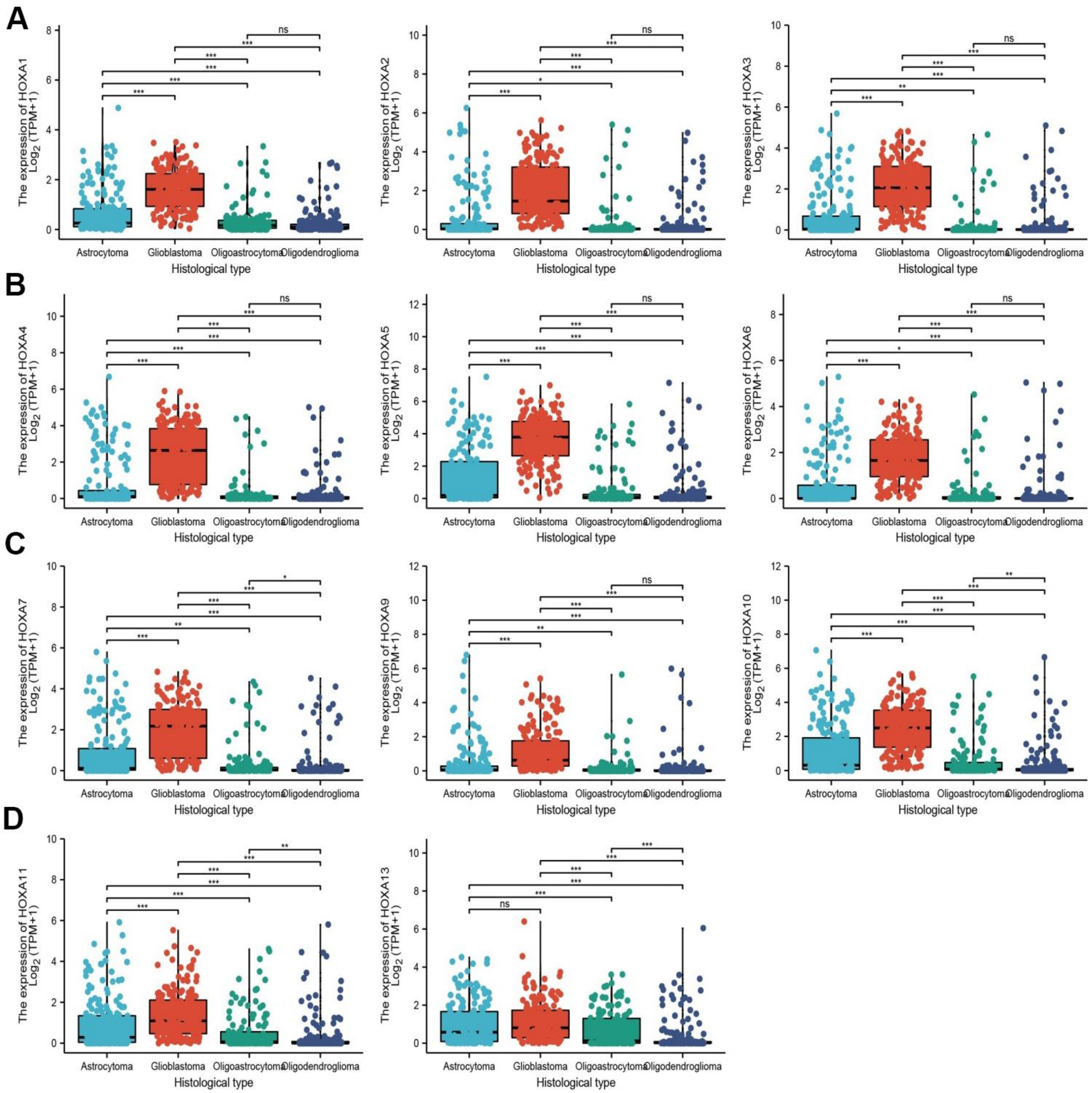

Supplementary Figure 1. The correlation between HOXAs and histological type in LGG. (**A**–**D**) Analysis correlation between HOXAs and histological type in LGG examine by TCGA database.

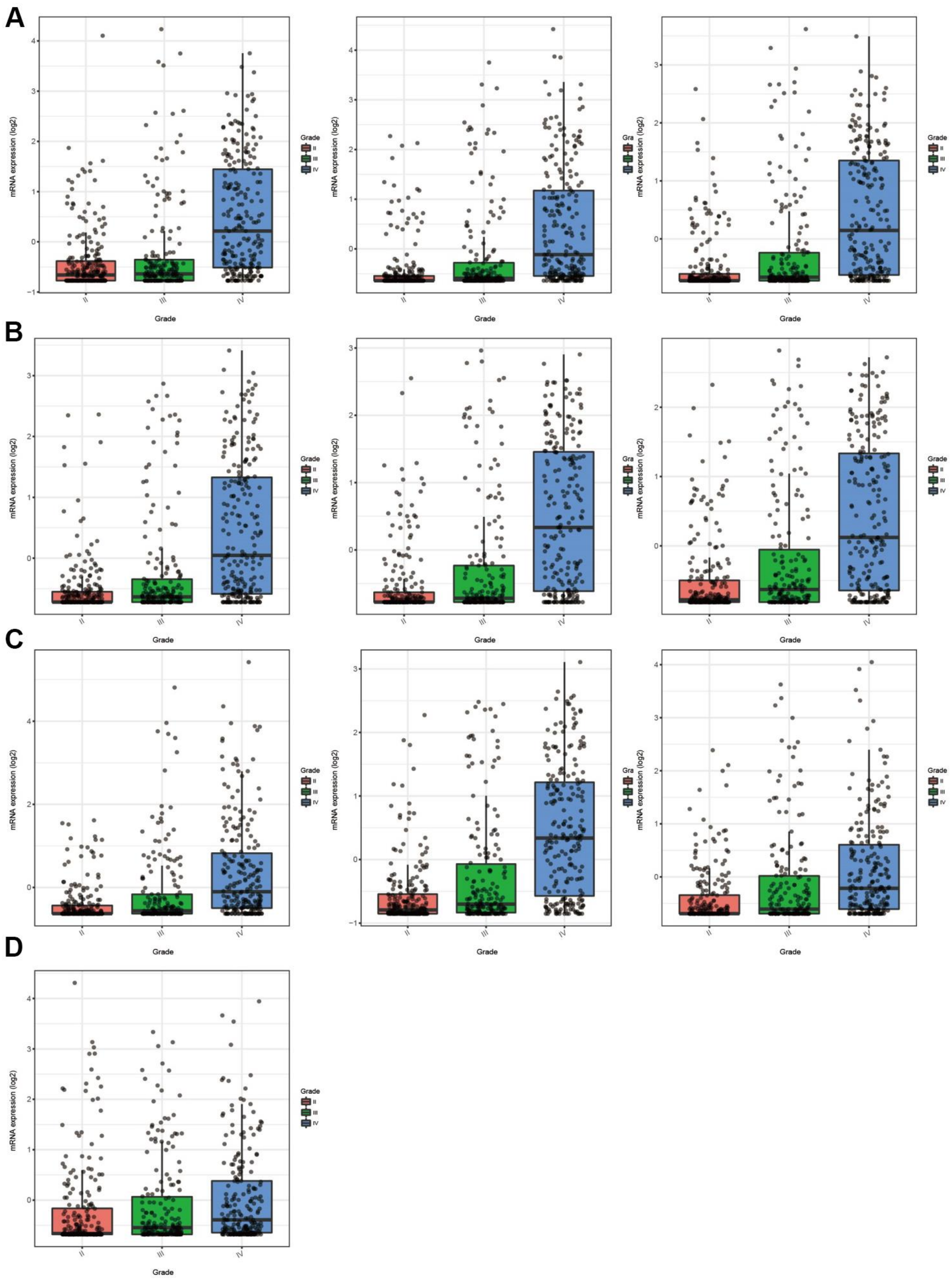

Supplementary Figure 2. The correlation between HOXAs and tumor grade in LGG. (**A**–**D**) The correlation between HOXAs and tumor grade in LGG by CGGA database.

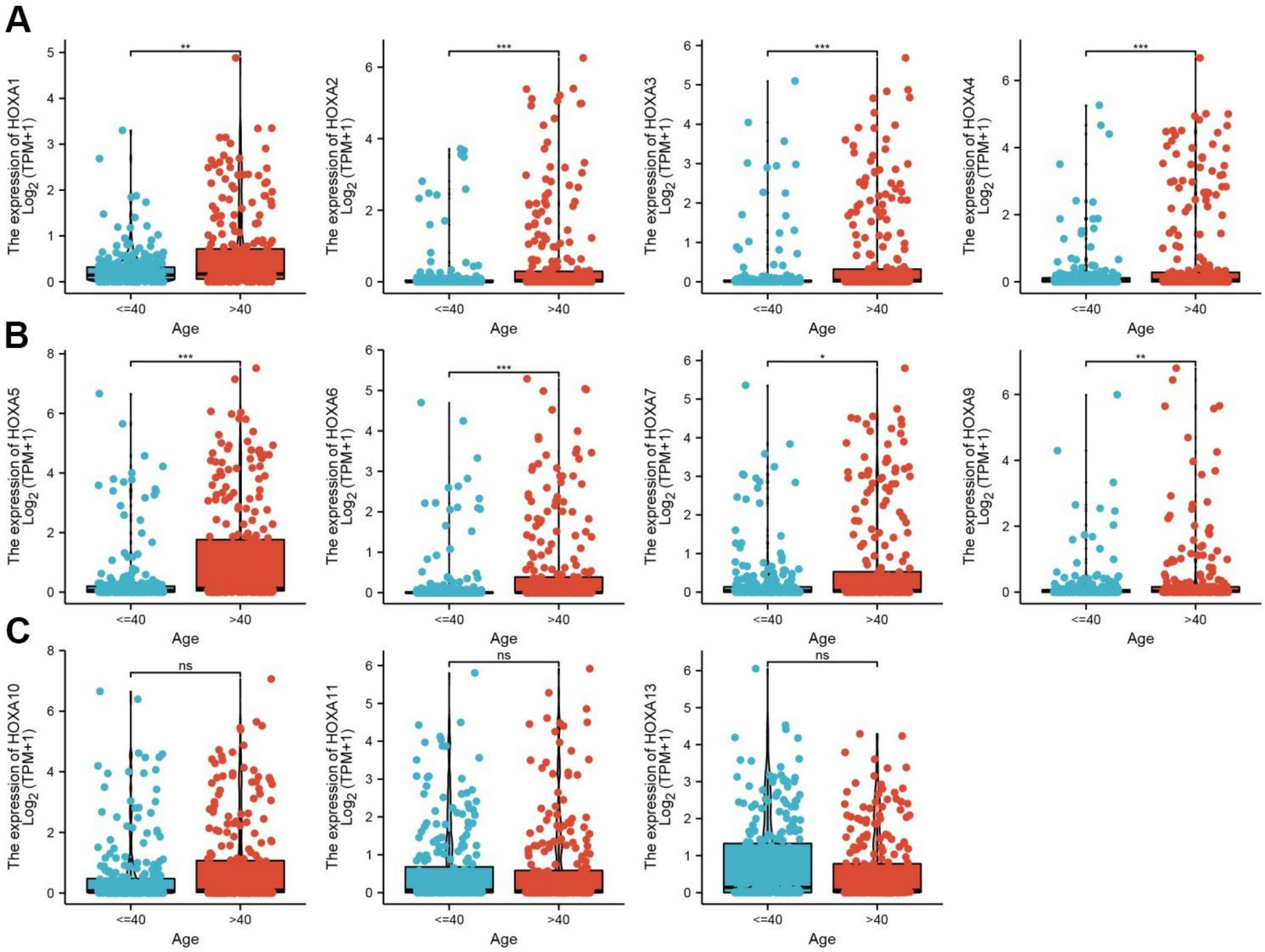

Supplementary Figure 3. The correlation between HOXAs and age in LGG. (**A**–**C**) The correlation between HOXAs and age in LGG by CGGA database.

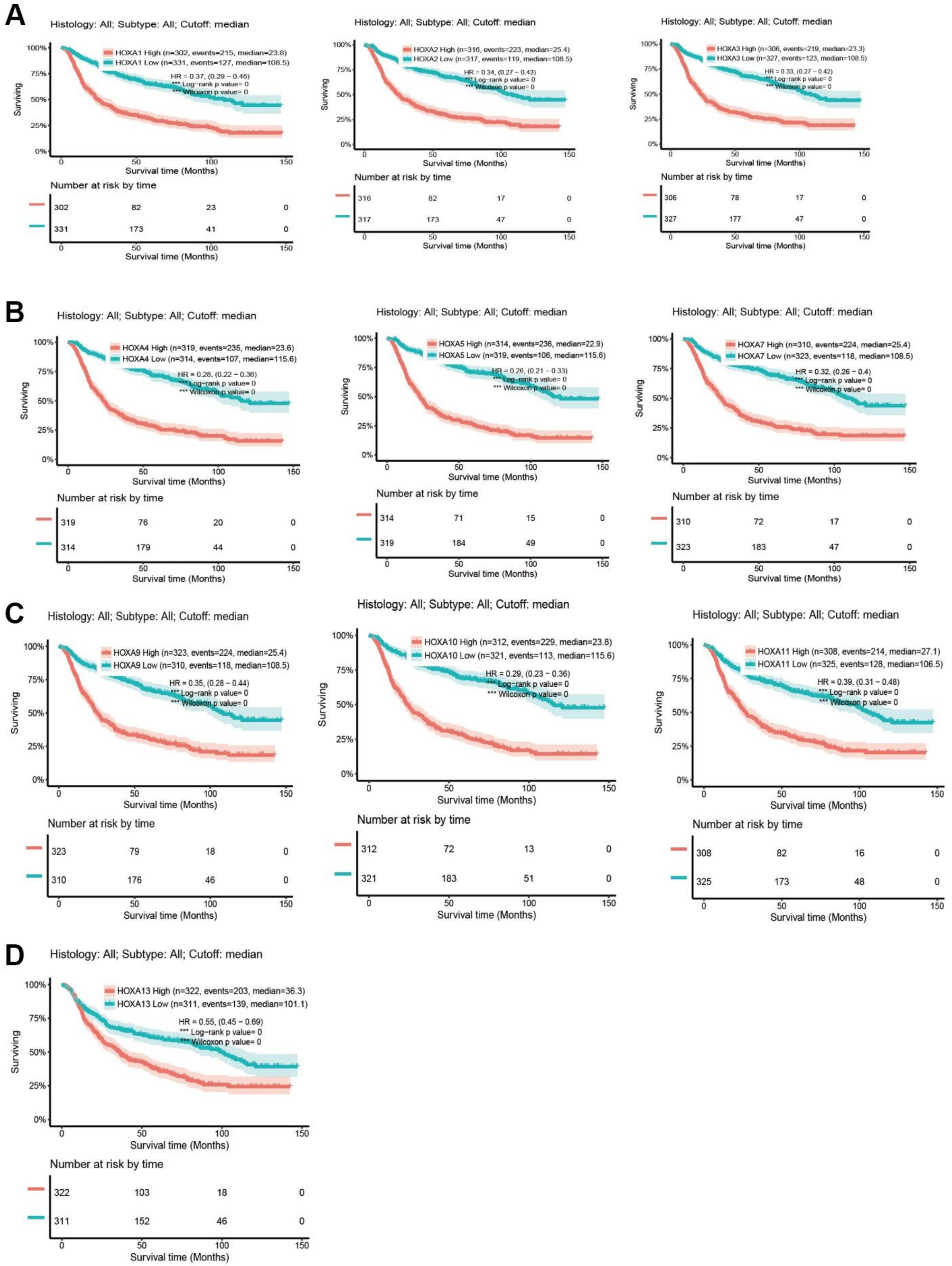

Supplementary Figure 4. Analysis the prognosis of HOXAs in LGG examine by CGGA database. (**A**–**D**) Analysis the prognosis of HOXAs in LGG examine by CGGA database.

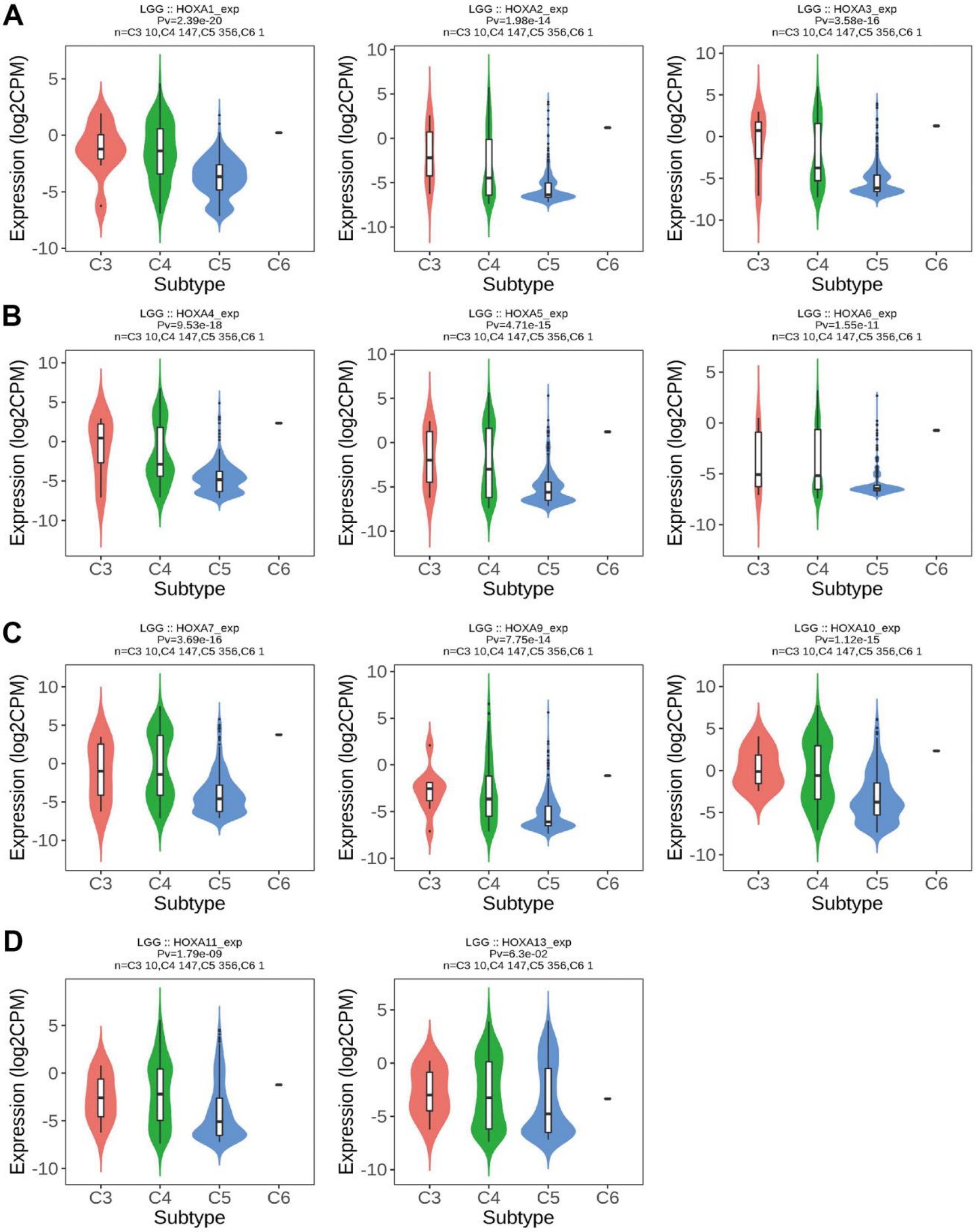

Supplementary Figure 5. The expression of HOXAs in immune subtype of LGG examine by TISIDB database. (**A**–**D**) The expression of HOXAs in immune subtype of LGG examine by TISIDB database.

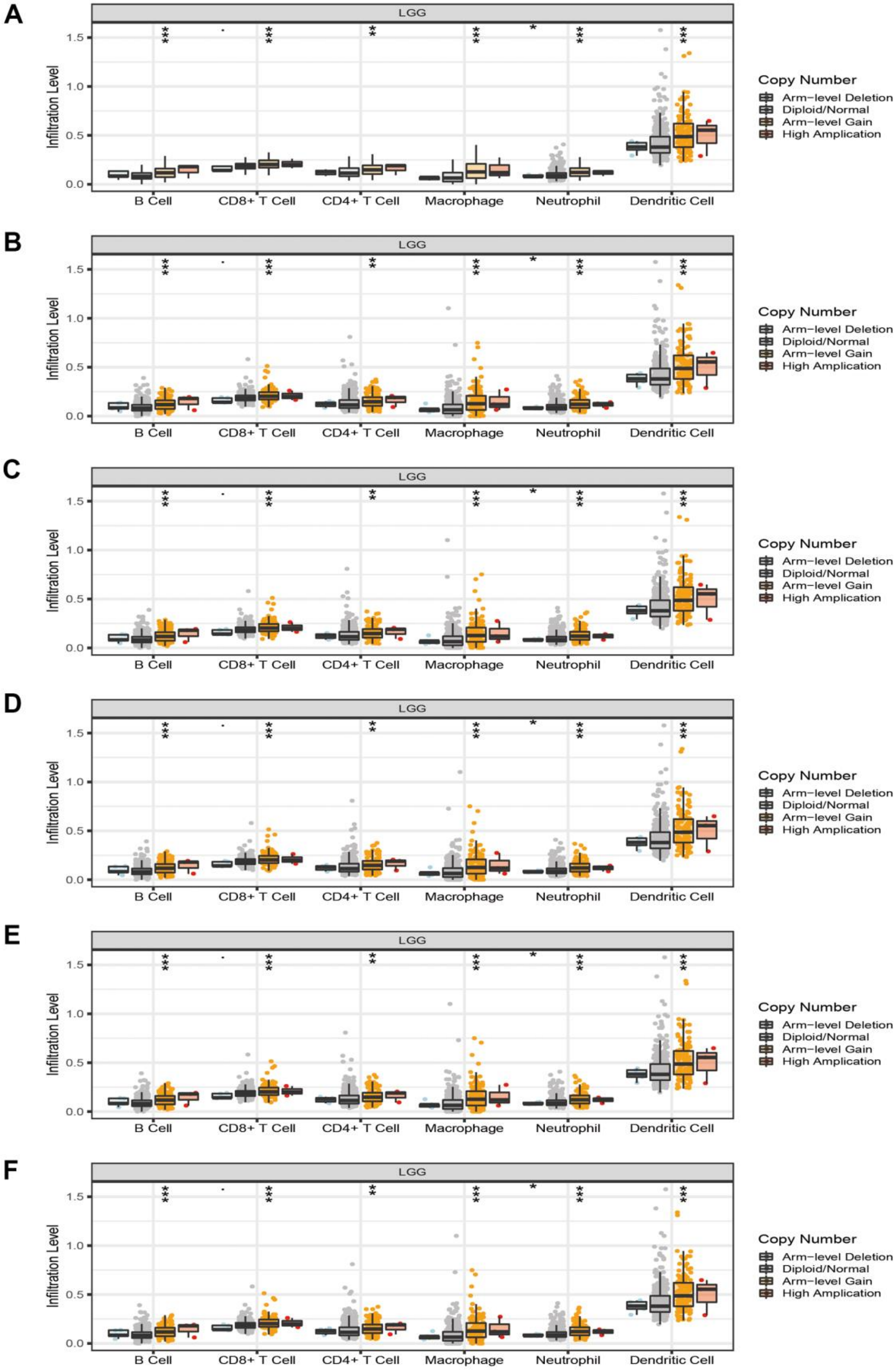

Supplementary Figure 6. Analysis the correlation between somatic copy number alterations of HOXAs and immune cell infiltration in LGG. (**A**–**F**) Analysis the correlation between somatic copy number alterations of HOXAs and immune cell infiltration levels in LGG based on TIMER database.

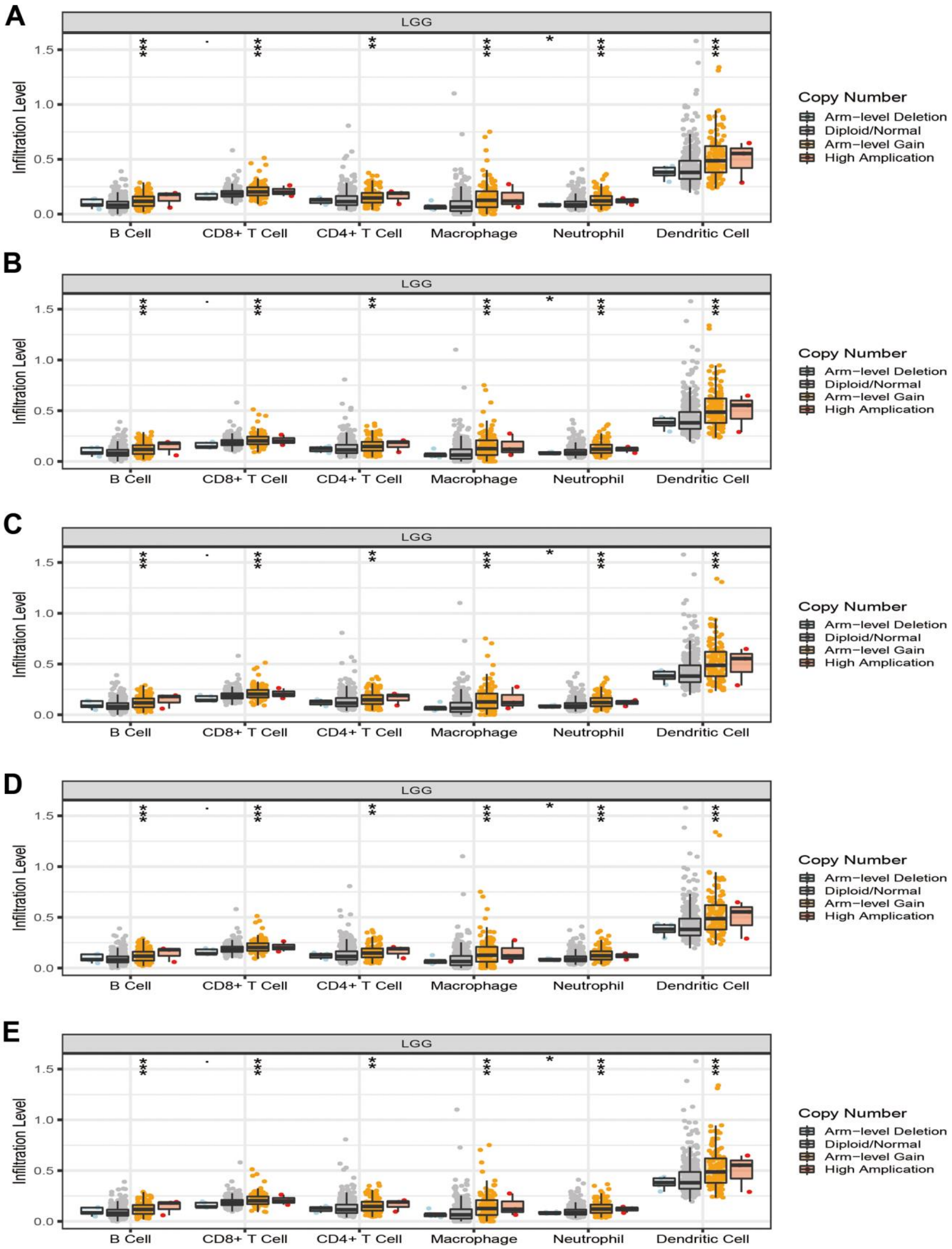

Supplementary Figure 7. Analysis the correlation between somatic copy number alterations of HOXAs and immune cell infiltration in LGG. (**A**–**E**) Analysis the correlation between somatic copy number alterations of HOXAs and immune cell infiltration levels in LGG based on TIMER database.

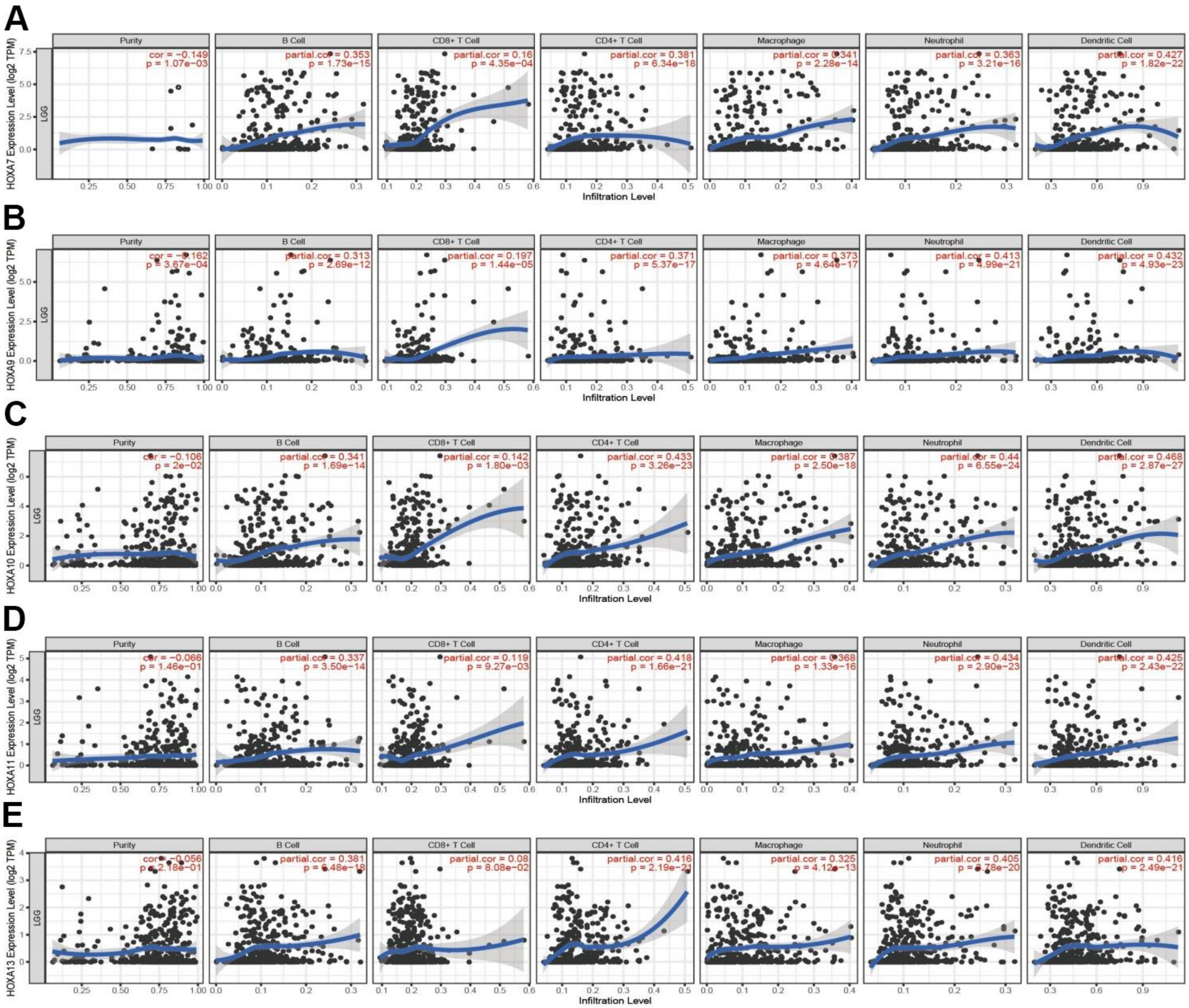

Supplementary Figure 8. Analysis the correlation between the HOXAs expression and immune cells infiltration. (**A**–**E**) The correlation between HOXAs expression and immune cells infiltration in LGG examine by the TIMER database. * P < 0.05, ** P < 0.01, *** P < 0.001.

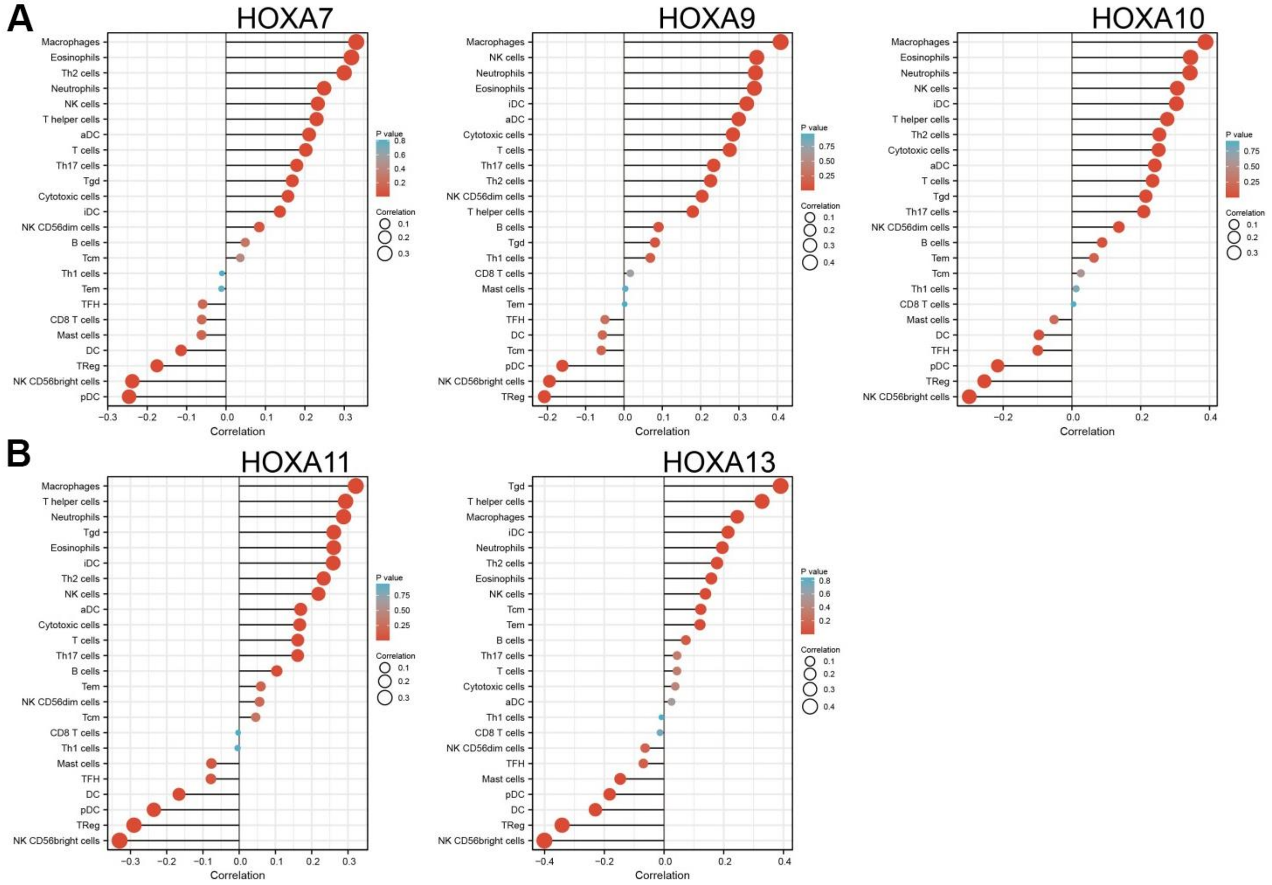

Supplementary Figure 9. Analysis the correlation between the HOXAs expression and immune cells infiltration. (**A**, **B**) The correlation between HOXAs expression and immune cells infiltration in LGG examine by the ssGSEA database. * P < 0.05, ** P < 0.01, *** P < 0.001.

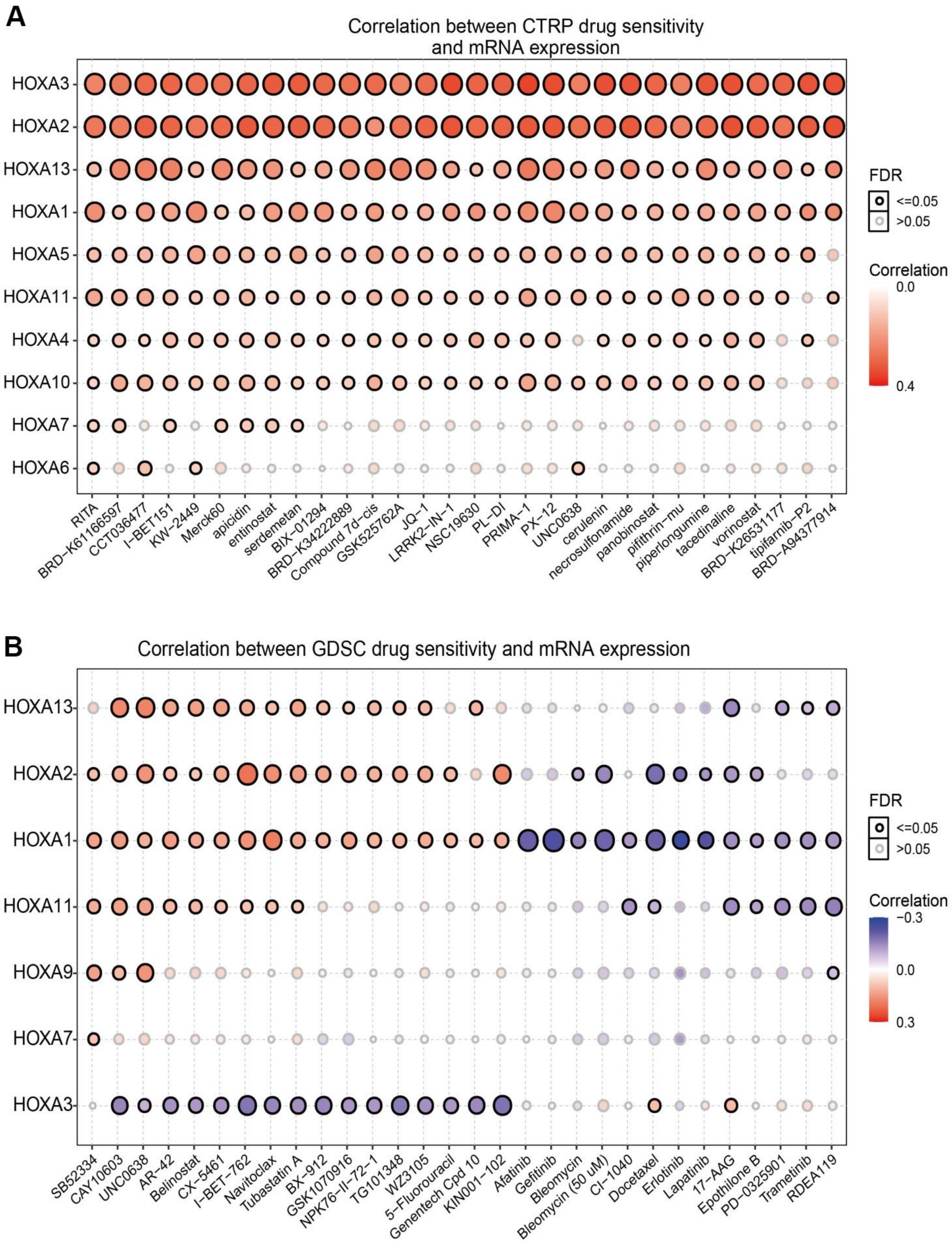

Supplementary Figure 10. The correlation between HOXAs expression and drug sensitivity. (**A**, **B**) The correlation between HOXAs expression and drug sensitivity by GDSC and CTRP database.

A

LncRNAs	miRNAs	mRNAs	FDR
SNHG12	hsa-miR-210-3p	HOXA1	2.27E-4
ZNF337-AS1	hsa-miR-210-3p	HOXA1	2.39E-2

LncRNAs	miRNAs	mRNAs	FDR
CTC-459F4.3	hsa-miR-142-3p	HOXA7	3.89E-5
HCG18	hsa-miR-196a-5p	HOXA7	2.93E-176
TRG-AS1	hsa-miR-196a-5p	HOXA7	3.07E-10

LncRNAs	miRNAs	mRNAs	FDR
LINC00665	hsa-miR-20a-5p	HOXA9	5.15E-28
RP5-1024G6.5	hsa-miR-20a-5p	HOXA9	2.26E-7
ZNF790-AS1	hsa-miR-20a-5p	HOXA9	1.62E-5
SNHG10	hsa-miR-145-5p	HOXA9	4.43E-5
FLJ37453	hsa-miR-20a-5p	HOXA9	4.74E-5
H19	hsa-miR-145-5p	HOXA9	5.78E-5
KDM4A-AS1	hsa-miR-145-5p	HOXA9	2.87E-4
SNHG12	hsa-miR-210-3p	HOXA9	6.97E-4
CTC-459F4.3	hsa-miR-142-3p	HOXA9	3.81E-2

B

LncRNAs	miRNAs	mRNAs	FDR
LINC00174	hsa-miR-26a-5p	HOXA5	4.91E-19
LINC00667	hsa-miR-19a-3p	HOXA5	2.07E-3
LINC00174	hsa-miR-26a-5p	HOXA5	1.84E-4

LncRNAs	miRNAs	mRNAs	FDR
LINC00665	hsa-miR-320a	HOXA10	3.92E-29
LIPE-AS1	hsa-miR-320a	HOXA10	5.17E-13
CTB-55O6.12	hsa-miR-320a	HOXA10	1.51E-7
SNHG12	hsa-miR-320a	HOXA10	8.43E-4
SNHG22	hsa-miR-204-5p	HOXA10	1.78E-3

LncRNAs	miRNAs	mRNAs	FDR
RP11-37B2.1	hsa-miR-124-3p	HOXA13	4.56E-9
KDM4A-AS1	hsa-miR-27a-5p	HOXA13	1.17E-6

Supplementary Figure 11. The ceRNA networks of HOXAs in LGG. (**A**, **B**) The ceRNA networks of HOXAs in LGG constructed by LnCeVar database.
