# Supplementary material for: Next-generation sequencing identifies HOXA6 as a novel oncogenic gene in low grade glioma
Source: Aging (Albany NY). 2022 Mar 29;14(6):2819–54. doi: 10.18632/aging.203977 (PMC9004573; doi:10.18632/aging.203977)
Supplement: Supplementary Table 1 [file aging-14-203977-s002.docx]

**Supplementary Table 1. Clinicopathological characteristics of LGG patients with differential HOXAs expression.**

| Characteristic | Low expression of HOXA1 | High expression of HOXA1 | p |
| --- | --- | --- | --- |
| n | 264 | 264 |  |
| WHO grade, n (%) |  |  | < 0.001 |
| G2 | 145 (31%) | 79 (16.9%) |  |
| G3 | 92 (19.7%) | 151 (32.3%) |  |
| IDH status, n (%) |  |  | < 0.001 |
| WT | 10 (1.9%) | 87 (16.6%) |  |
| Mut | 254 (48.4%) | 174 (33.1%) |  |
| 1p/19q codeletion, n (%) |  |  | < 0.001 |
| codel | 127 (24.1%) | 44 (8.3%) |  |
| non-codel | 137 (25.9%) | 220 (41.7%) |  |
| Age, median (IQR) | 39 (32, 51) | 42 (32, 55) | 0.117 |

| Characteristic | Low expression of HOXA2 | High expression of HOXA2 | p |
| --- | --- | --- | --- |
| n | 264 | 264 |  |
| WHO grade, n (%) |  |  | 0.002 |
| G2 | 131 (28.1%) | 93 (19.9%) |  |
| G3 | 106 (22.7%) | 137 (29.3%) |  |
| IDH status, n (%) |  |  | < 0.001 |
| WT | 17 (3.2%) | 80 (15.2%) |  |
| Mut | 246 (46.9%) | 182 (34.7%) |  |
| 1p/19q codeletion, n (%) |  |  | < 0.001 |
| codel | 114 (21.6%) | 57 (10.8%) |  |
| non-codel | 150 (28.4%) | 207 (39.2%) |  |
| Age, median (IQR) | 38 (31, 47.25) | 44 (33, 57) | < 0.001 |

| Characteristic | Low expression of HOXA3 | High expression of HOXA3 | p |
| --- | --- | --- | --- |
| n | 264 | 264 |  |
| WHO grade, n (%) |  |  | < 0.001 |
| G2 | 142 (30.4%) | 82 (17.6%) |  |
| G3 | 97 (20.8%) | 146 (31.3%) |  |
| IDH status, n (%) |  |  | < 0.001 |
| WT | 18 (3.4%) | 79 (15%) |  |
| Mut | 245 (46.7%) | 183 (34.9%) |  |
| 1p/19q codeletion, n (%) |  |  | < 0.001 |
| codel | 116 (22%) | 55 (10.4%) |  |
| non-codel | 148 (28%) | 209 (39.6%) |  |
| Age, median (IQR) | 37 (30, 48) | 44 (35, 56) | < 0.001 |

| Characteristic | Low expression of HOXA4 | High expression of HOXA4 | p |
| --- | --- | --- | --- |
| n | 264 | 264 |  |
| WHO grade, n (%) |  |  | < 0.001 |
| G2 | 136 (29.1%) | 88 (18.8%) |  |
| G3 | 100 (21.4%) | 143 (30.6%) |  |
| IDH status, n (%) |  |  | < 0.001 |
| WT | 18 (3.4%) | 79 (15%) |  |
| Mut | 245 (46.7%) | 183 (34.9%) |  |
| 1p/19q codeletion, n (%) |  |  | < 0.001 |
| codel | 137 (25.9%) | 34 (6.4%) |  |
| non-codel | 127 (24.1%) | 230 (43.6%) |  |
| Age, median (IQR) | 40 (32, 51) | 41 (32, 55) | 0.129 |

| Characteristic | Low expression of HOXA5 | High expression of HOXA5 | p |
| --- | --- | --- | --- |
| n | 264 | 264 |  |
| WHO grade, n (%) |  |  | < 0.001 |
| G2 | 141 (30.2%) | 83 (17.8%) |  |
| G3 | 94 (20.1%) | 149 (31.9%) |  |
| IDH status, n (%) |  |  | < 0.001 |
| WT | 17 (3.2%) | 80 (15.2%) |  |
| Mut | 246 (46.9%) | 182 (34.7%) |  |
| 1p/19q codeletion, n (%) |  |  | < 0.001 |
| codel | 132 (25%) | 39 (7.4%) |  |
| non-codel | 132 (25%) | 225 (42.6%) |  |
| Age, median (IQR) | 38 (31, 50) | 43 (34, 55) | < 0.001 |

| Characteristic | Low expression of HOXA7 | High expression of HOXA7 | p |
| --- | --- | --- | --- |
| n | 264 | 264 |  |
| WHO grade, n (%) |  |  | < 0.001 |
| G2 | 133 (28.5%) | 91 (19.5%) |  |
| G3 | 100 (21.4%) | 143 (30.6%) |  |
| IDH status, n (%) |  |  | < 0.001 |
| WT | 21 (4%) | 76 (14.5%) |  |
| Mut | 243 (46.3%) | 185 (35.2%) |  |
| 1p/19q codeletion, n (%) |  |  | < 0.001 |
| codel | 137 (25.9%) | 34 (6.4%) |  |
| non-codel | 127 (24.1%) | 230 (43.6%) |  |
| Age, median (IQR) | 39.5 (31, 50) | 41.5 (33, 56) | 0.021 |

| Characteristic | Low expression of HOXA9 | High expression of HOXA9 | p |
| --- | --- | --- | --- |
| n | 264 | 264 |  |
| WHO grade, n (%) |  |  | < 0.001 |
| G2 | 137 (29.3%) | 87 (18.6%) |  |
| G3 | 101 (21.6%) | 142 (30.4%) |  |
| IDH status, n (%) |  |  | < 0.001 |
| WT | 19 (3.6%) | 78 (14.9%) |  |
| Mut | 243 (46.3%) | 185 (35.2%) |  |
| 1p/19q codeletion, n (%) |  |  | < 0.001 |
| codel | 127 (24.1%) | 44 (8.3%) |  |
| non-codel | 137 (25.9%) | 220 (41.7%) |  |
| Age, median (IQR) | 39 (31, 52) | 43 (33, 54) | 0.061 |

| Characteristic | Low expression of HOXA10 | High expression of HOXA10 | p |
| --- | --- | --- | --- |
| n | 264 | 264 |  |
| WHO grade, n (%) |  |  | < 0.001 |
| G2 | 138 (29.6%) | 86 (18.4%) |  |
| G3 | 95 (20.3%) | 148 (31.7%) |  |
| IDH status, n (%) |  |  | < 0.001 |
| WT | 26 (5%) | 71 (13.5%) |  |
| Mut | 237 (45.1%) | 191 (36.4%) |  |
| 1p/19q codeletion, n (%) |  |  | < 0.001 |
| codel | 145 (27.5%) | 26 (4.9%) |  |
| non-codel | 119 (22.5%) | 238 (45.1%) |  |
| Age, median (IQR) | 39.5 (32, 50) | 41 (32.75, 55) | 0.093 |

| Characteristic | Low expression of HOXA11 | High expression of HOXA11 | p |
| --- | --- | --- | --- |
| n | 264 | 264 |  |
| WHO grade, n (%) |  |  | < 0.001 |
| G2 | 135 (28.9%) | 89 (19.1%) |  |
| G3 | 100 (21.4%) | 143 (30.6%) |  |
| IDH status, n (%) |  |  | < 0.001 |
| WT | 30 (5.7%) | 67 (12.8%) |  |
| Mut | 234 (44.6%) | 194 (37%) |  |
| 1p/19q codeletion, n (%) |  |  | < 0.001 |
| codel | 153 (29%) | 18 (3.4%) |  |
| non-codel | 111 (21%) | 246 (46.6%) |  |
| Age, median (IQR) | 42 (33, 53) | 39 (32, 52) | 0.240 |

| Characteristic | Low expression of HOXA13 | High expression of HOXA13 | p |
| --- | --- | --- | --- |
| n | 264 | 264 |  |
| WHO grade, n (%) |  |  | 0.006 |
| G2 | 129 (27.6%) | 95 (20.3%) |  |
| G3 | 108 (23.1%) | 135 (28.9%) |  |
| IDH status, n (%) |  |  | 0.0461 |
| WT | 45 (8.6%) | 52 (9.9%) |  |
| Mut | 219 (41.7%) | 209 (39.8%) |  |
| 1p/19q codeletion, n (%) |  |  | < 0.001 |
| codel | 160 (30.3%) | 11 (2.1%) |  |
| non-codel | 104 (19.7%) | 253 (47.9%) |  |
| Age, median (IQR) | 42 (33, 54) | 39 (31, 51) | 0.039 |
